# Host-microbiome determinants of ready-to-use supplemental food efficacy in acute childhood malnutrition

**DOI:** 10.1172/jci.insight.188993

**Published:** 2025-07-22

**Authors:** Zehra Jamil, Gabriel F. Hanson, Junaid Iqbal, G. Brett Moreau, Najeeha Talat Iqbal, Sheraz Ahmed, Aneeta Hotwani, Furqan Kabir, Fayaz Umrani, Kamran Sadiq, Kumail Ahmed, Indika Mallawaarachchi, Jennie Z. Ma, Fatima Aziz, S. Asad Ali, Sean R. Moore

**Affiliations:** 1 Department of Biological & Biomedical Sciences and; 2 Department of Community & Health Sciences, Aga Khan University, Karachi, Pakistan.; 3 Department of Biomedical Engineering, University of Virginia, Charlottesville, Virginia, USA.; 4 Department of Pediatrics & Child Health, Aga Khan University, Karachi, Pakistan.; 5 Division of Infectious Diseases and International Health, Department of Medicine, University of Virginia School of Medicine, Charlottesville, Virginia, USA.; 6 Department of Public Health Sciences, University of Virginia, Charlottesville, Virginia, USA.; 7 Division of Gastroenterology, Hepatology and Nutrition, Cincinnati Children’s Hospital, Cincinnati, Ohio, USA.

**Keywords:** Gastroenterology, Microbiology, Bioinformatics

## Abstract

**Background:**

Ready-to-use supplemental foods (RUSF) are energy-dense meals used to treat moderate and severe acute childhood malnutrition. Weight recovery with RUSF is heterogeneous, therefore we investigated whether environmental enteric dysfunction (EED), systemic inflammation, and gut microbiota predict RUSF response.

**Methods:**

We followed nutritional status and RUSF outcomes in a rural birth cohort of 416 Pakistani infants. Acha Mum, a chickpea-based RUSF, was administered daily for 8 weeks to children who developed wasting (weight-for-length Z-score <–2).

**Results:**

Of 187 treated with RUSF, 112 showed no immediate improvement in weight-for-age. Machine learning identified nine biomarkers that collectively predicted RUSF response with 73% accuracy. Gut microbiome composition before and after supplementation predicted response with 93% and 98% accuracy, respectively. Responders showed microbiome restructuring, with increased growth-associated taxa and reduced Gammaproteobacteria relative to nonresponders. A subset of extreme nonresponders—whose microbiome profiles resembled those of responders—displayed markedly abnormal biomarkers of inflammation, suggesting adverse host factors constrain gut microbiota benefits for RUSF efficacy.

**Conclusion:**

EED, systemic inflammation, and gut microbiota predict acute nutritional responses to Acha Mum, setting the stage for precision use of RUSF and adjunctive therapies in addressing the global burden of childhood malnutrition in low- and middle-income countries.

## Abstract 2

The response to nutritional intervention in children at risk of developing environmental enteropathy is heralded by underlying gut microbiome signatures and heightened serum and fecal inflammatory biomarkers.

## Graphical abstract

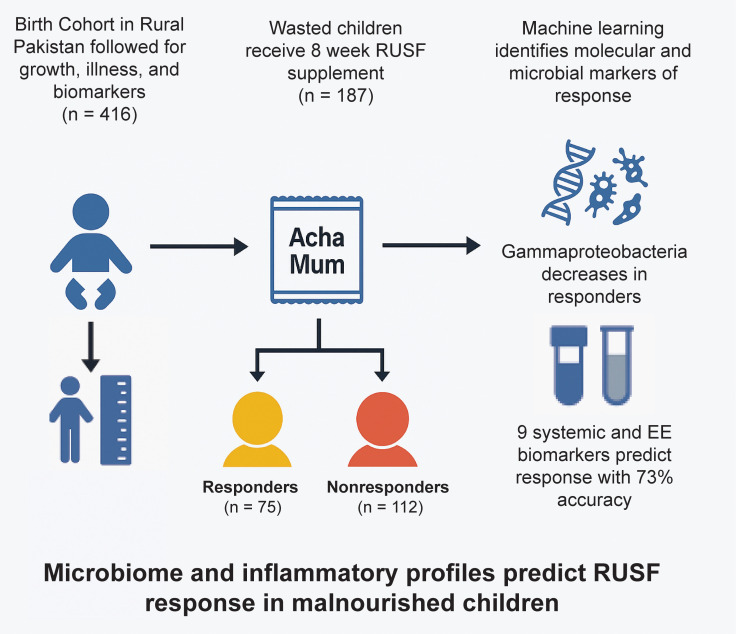

## Introduction

The 2025 WHO Global Nutrition Targets call for dramatic and overdue reductions in the global burden of childhood wasting (acute undernutrition) and stunting (chronic undernutrition) (1). Persistent health and economic disparities in low- and middle-income countries (LMICs) continue to impede progress toward these goals (2); hence, the timely application of effective public health interventions as well as an improved understanding of the mechanisms underlying childhood undernutrition in LMICs will be crucial to success (3). Ready-to-use supplemental foods (RUSFs) have emerged as a key strategy for the prevention and treatment of moderate and severe acute malnutrition (moderate acute malnutrition [MAM], weight-for-length *z* [WLZ] score less than –2 but greater than –3; severe acute malnutrition [SAM], WLZ < –3 SD) in community-based settings. Efforts to encourage the local production of RUSF, enhance efficacy and adherence, and reduce gastrointestinal side effects (e.g., vomiting) are on the rise (4); however, RUSF interventions continue to show inconsistent efficacy and durability for growth recovery (5). Optimizing RUSF composition, duration, monitoring, and adjunctive therapies to maximize nutritional benefit is currently constrained by a lack of precision tools to identify those children least likely to respond.

Childhood growth faltering in LMICs is closely linked to environmental enteric dysfunction (EED), a subclinical enteropathy characterized by gut and systemic inflammation, intestinal barrier dysfunction, and malabsorption (6). It is not yet clear to what extent EED impairs the efficacy of RUSF in childhood wasting. Intensive nutritional and WASH (water, sanitation, and hygiene) interventions against childhood stunting have produced only modest benefits, raising concerns that the derangements in gut structure and function seen in EED undermine these strategies (7). Recently, causal links between gut microbiota and early childhood undernutrition have been identified, leading to investigations exploring microbiota-directed complementary foods (MDCF) (8, 9). Although the role of EED in stunting pathways is increasingly appreciated, the degree to which EED impairs the efficacy of RUSF and MDCF is not yet understood.

Building on these observations, we explored whether host-microbiome factors influence RUSF response in acutely undernourished children to address the following questions. (a) To what extent do EED and systemic inflammation undermine the efficacy of RUSF? (b) Do baseline differences in gut microbiota prior to RUSF administration predict clinical outcomes? (c) Do RUSF-driven shifts in gut microbial communities differentiate responders from nonresponders? Here, we report findings from secondary analyses of the Study of Environmental Enteropathy and Malnutrition-Pakistan (SEEM-Pakistan), in which 187 children with recalcitrant wasting received a chickpea-based RUSF. We found that: (a) biomarkers of systemic inflammation, EED, and nutritional status (fecal myeloperoxidase [MPO] and neopterin [NEO]; serum prealbumin, glucagon-like peptide-2, and CRP; and urine claudin-15 and creatinine) collectively predict RUSF response; (b) pre-RUSF microbiome composition predicts RUSF response; and (c) RUSF expands the relative abundance of Negativicutes (including *Veillonella* chickpea fermenters) in wasted infants, with responders displaying greater relative increases in Negativicutes and Clostridia*,* and decreases in Gammaproteobacteria*,* relative to nonresponders.

## Results

### Participant characteristics.

This study includes participants selected from the parent study, SEEM, in Pakistan in which 416 infants were enrolled as cases (*n* = 365) and controls (*n* = 51) from a rural setting. Infants (0–1 month old) were screened by monitoring their weight and length monthly up to 6 months and were then classified as either well-nourished controls (WLZ ≥ 0, length-for-age *z* [LAZ] ≥-1, *n* = 51) or undernourished controls (wasted with WLZ ≤ –2, *n* = 365) based on at least 2 consecutive months of anthropometry. Of the 365 controls, wasted infants (WLZ < –2, *n* = 187) whose growth did not improve after their primary caregiver participated in an intensive nutritional education intervention were provided Acha Mum at the age of 9 months, a locally produced chickpea-based RUSF, daily for 8 weeks. The blood, fecal, and urine samples were collected prior to the initiation of RUSF, to determine EED biomarkers. These baseline characteristics and biomarkers are summarized in Table 1. Maternal and paternal BMI, as well as infant anthropometrics (0–1 month of age), were significantly lower in controls (*P* < 0.05), with differences that became even more pronounced by 9 months of age (*P* < 0.05). Breastfeeding patterns were similar between groups during the first 6 months of life. Of these 187 controls, 112 responded (41%), while 75 (59%) were classified as nonresponders as assessed by WLZ 1-week after intervention (10). To select 60 infants for this substudy (of 187 controls in SEEM), we calculated weight-for-age (WAZ) velocity (ΔWAZ/month) during the 8-week intervention rather than WLZ velocity, given that changes in infant length are more challenging to reliably measure and detect over an 8-week nutritional intervention relative to changes in infant weight.

**Figure 1:**
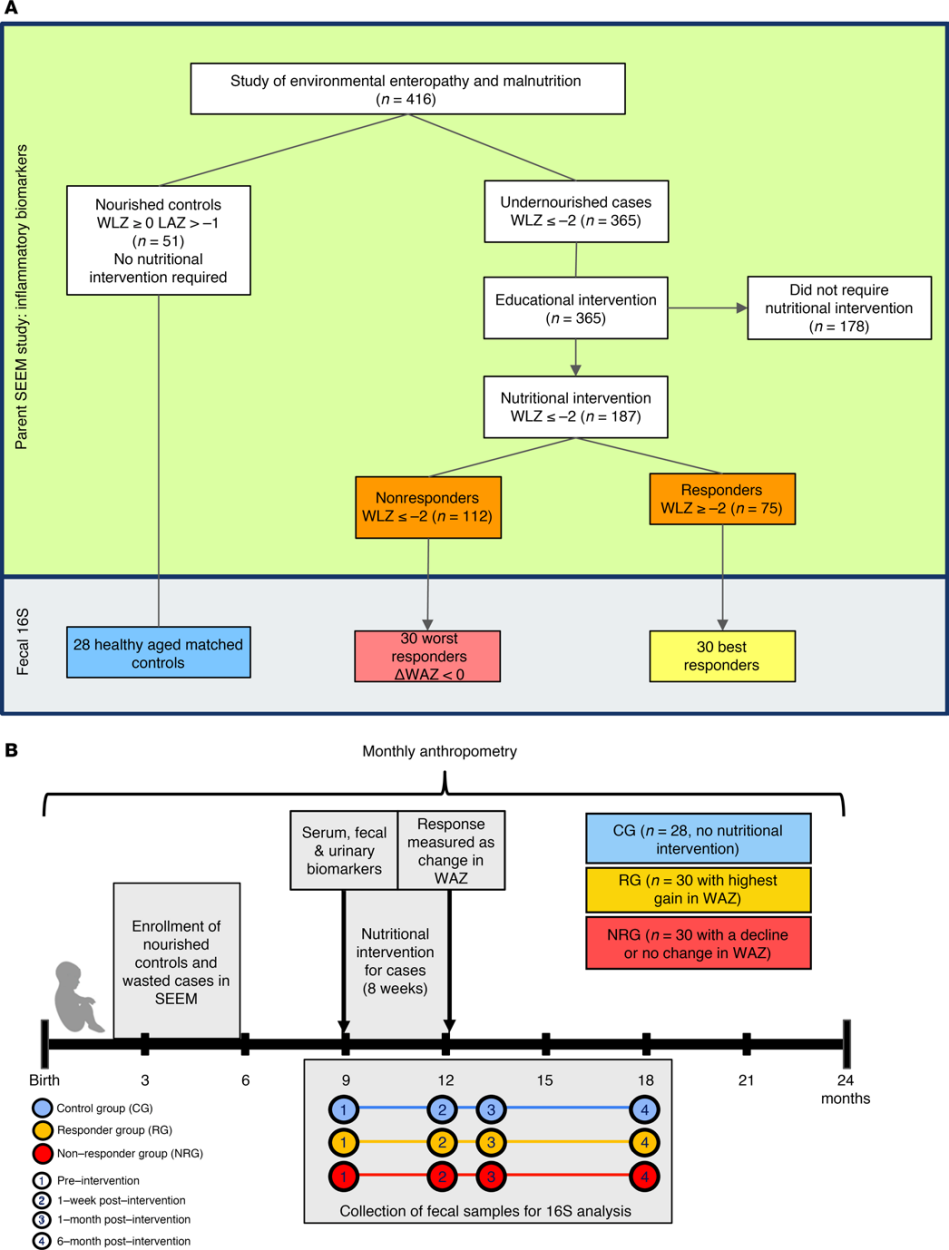
Overview of study design. (**A**) Flow diagram of study population and protocols. (**B**) Infogram of study timelines and sample collection.

Based on ΔWAZ, 30 best responders with ΔWAZ > 0.5/month and 30 worst responses with ΔWAZ ≤ 0/month were selected in addition to 28 controls (of the 51 controls from the parent study), as they lived in the same settings with similar socioeconomic statuses (Figure 1A). We performed fecal 16S sequencing of samples collected at 4 time points: (preintervention at 9 months, 1 week after intervention at 12 months, 1 month after intervention [13 months], and 6 months after intervention [18 months]) as shown in Figure 1B. Relabeling the controls based on their response, the baseline characteristics of the 88 participants in this sub-study are summarized in Supplemental Tables 1 and 2 (supplemental material available online with this article; https://doi.org/10.1172/jci.insight.188993DS1). These selected 88 participants were a true representation of the larger SEEM cohort (Supplemental Table 3).

### Ponderal growth responses of selected participants to RUSF administration.

**Figure 2:**
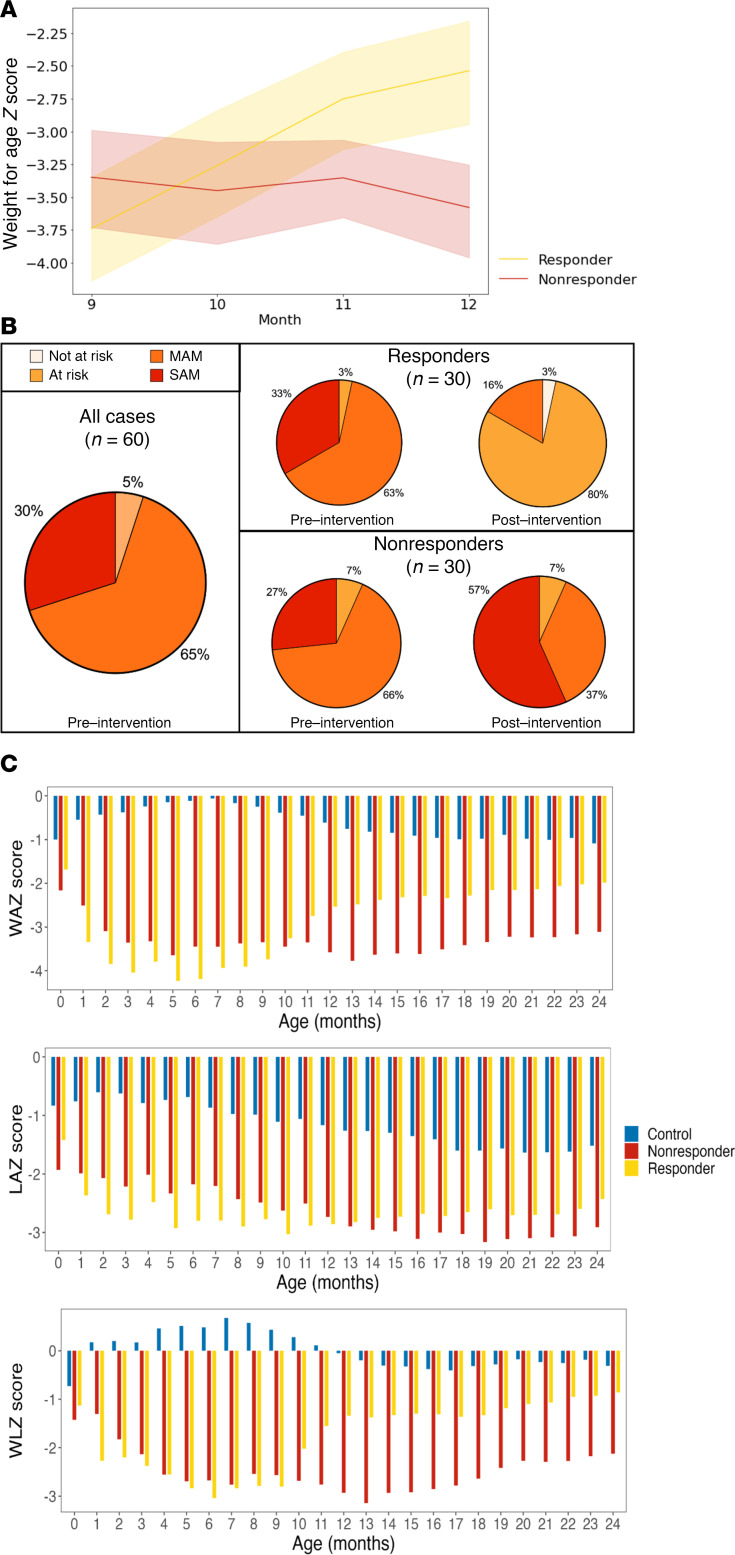
Children selected for gut microbiome analysis show diverging yet durable responses to RUSF. (**A**) Mean change in WAZ score per month during RUSF. (**B**) Nutritional status of responders (*n* = 30) and nonresponders (*n* = 30) with 16S data before and after RUSF. (**C**) On average, responders had worse ponderal growth prior to RUSF but showed persistent growth improvements in response to RUSF as measured by weight-for-age *z* scores, length-for-age *z* scores, and weight-for-length *z* scores. Age- and region-matched well-nourished control cohort shown in blue (*n* = 28).

Regarding anthropometrics, 30 best responders (RUSF-R) demonstrated a mean change in WAZ of 0.413 over the course of the intervention (Figure 2A and Table 2). RUSF-R showed significant improvements in overall nutritional status; SAM declined from 33% to 0%, and MAM declined from 63% to 20% (Figure 2B). In contrast, the 30 children with the highest decline in WAZ during the course of intervention (RUSF-NR) demonstrated either no improvements in WAZ or a decline, with a mean monthly WAZ velocity over the intervention of –0.081. The proportion of children with SAM increased in the RUSF-NR from 27% at baseline to 57% postintervention. WAZ and WLZ scores were slightly lower in RUSF-R at baseline, indicating RUSF response was neither due to better nutritional status at baseline nor regression to the mean. Of note, RUSF-R were less wasted on their first anthropometrics (0 to 1 month) than RUSF-NR (Supplemental Table 1). All other baseline indices were similar between groups, including RUSF adherence and illness burden (Supplemental Figure 1A).

We tracked growth trajectories in RUSF-R and RUSF-NR beyond the completion of supplementation (Figure 2C). Improvements in WAZ and WLZ continued in RUSF-R after the intervention was completed, persisting until the end of follow-up at 24 months, indicating a durable RUSF effect. RUSF-NR did not respond immediately to nutritional intervention; however, we noted improvements in ponderal growth beginning at age 13 months (Table 2). Length-for-age *z* (LAZ) scores remained largely unchanged in both RUSF-R and RUSF-NR over the course of intervention and during follow-up. In controls who did not require nutritional intervention, anthropometric indices gradually declined during follow-up. Taken together, this suggests that RUSF was ultimately beneficial even for RUSF-NR.

### Serum, urine, and fecal biomarkers distinguish wasted children from controls.

**Figure 3:**
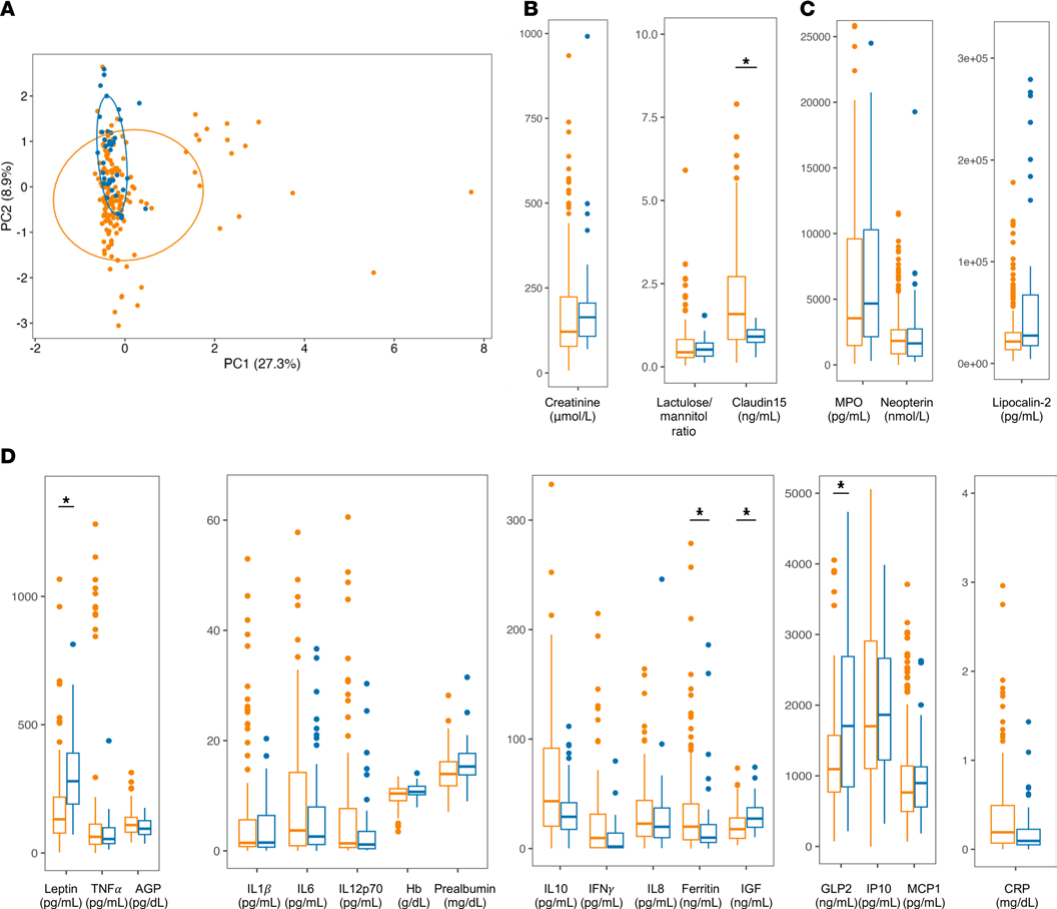
Malnourished children exhibit increased systemic inflammation compared with healthy age-matched controls. (**A**) PCA of biomarker profiles from malnourished children (*n* = 148) and healthy aged-matched children (*n* = 39). (**B**–**D**) Urine (**B**), fecal (**C**), and serum biomarker/cytokine (**D**) levels from children at 9 months of age prior to RUSF in the SEEM cohort. *P < 0.05. Wilcoxin’s rank-sum test with Benjamini Hochburg corrections.

We hypothesized baseline growth, inflammation, and EED biomarkers would be distinct in wasted infants compared with well-nourished controls. These biomarker data available from the entire SEEM cohort (*n* = 235) were compared using Principal Component Analysis (PCA) to identify global group differences (Figure 3A). Our model was able to separate controls and controls along Principal Component 2 (PC2), with variation driven by biomarkers of growth and nutritional status (IGF1, prealbumin, GLP2, and leptin) as well as acute phase proteins C-reactive protein (CRP) and Alpha-1–acid glycoprotein (AGP) (Supplemental Figure 2A). Control samples clustered tightly, whereas controls displayed a more heterogeneous distribution along PC1, with a subset of participants designated as controls regardless of nutritional status. PC1 represented most of the variation within the model, primarily driven by inflammatory cytokines such as IL-1β, IFN-γ, and TNF-α.

The PCA of the 88 study participants selected for this RUSF subanalysis (30 RUSF-R, 30 RUSF-NR, and 28 controls) showed a similar pattern of clustering (Supplemental Figure 2B), indicating that biomarker profiles from these subgroups were broadly representative of the total cohort.

PCA findings were confirmed by univariate analyses. Growth and nutritional status biomarkers, including IGF1, prealbumin, GLP2, and leptin, were significantly elevated in control samples compared with controls (Figure 3, B–D). Controls displayed evidence of systemic inflammation, with significantly higher concentrations of inflammatory biomarkers such as CRP, IL-6, IFN-γ, TNF-α, and IL-1β. Notably, the antiinflammatory cytokine IL-10 was higher in controls than in controls, suggesting feedback regulation. In addition, the urinary excretion of the intestinal tight junction protein claudin-15 was significantly higher in controls, consistent with previous associations of urinary claudin-15 with decreased gut barrier function observed in EED (11, 12). Fecal markers of increased intestinal inflammation were similar across controls and controls: fecal MPO and NEO levels were similar between groups, and fecal lipocalin-2 (LCN2) was elevated in controls compared with controls.

### Preintervention biomarkers predict RUSF response.

**Figure 4:**
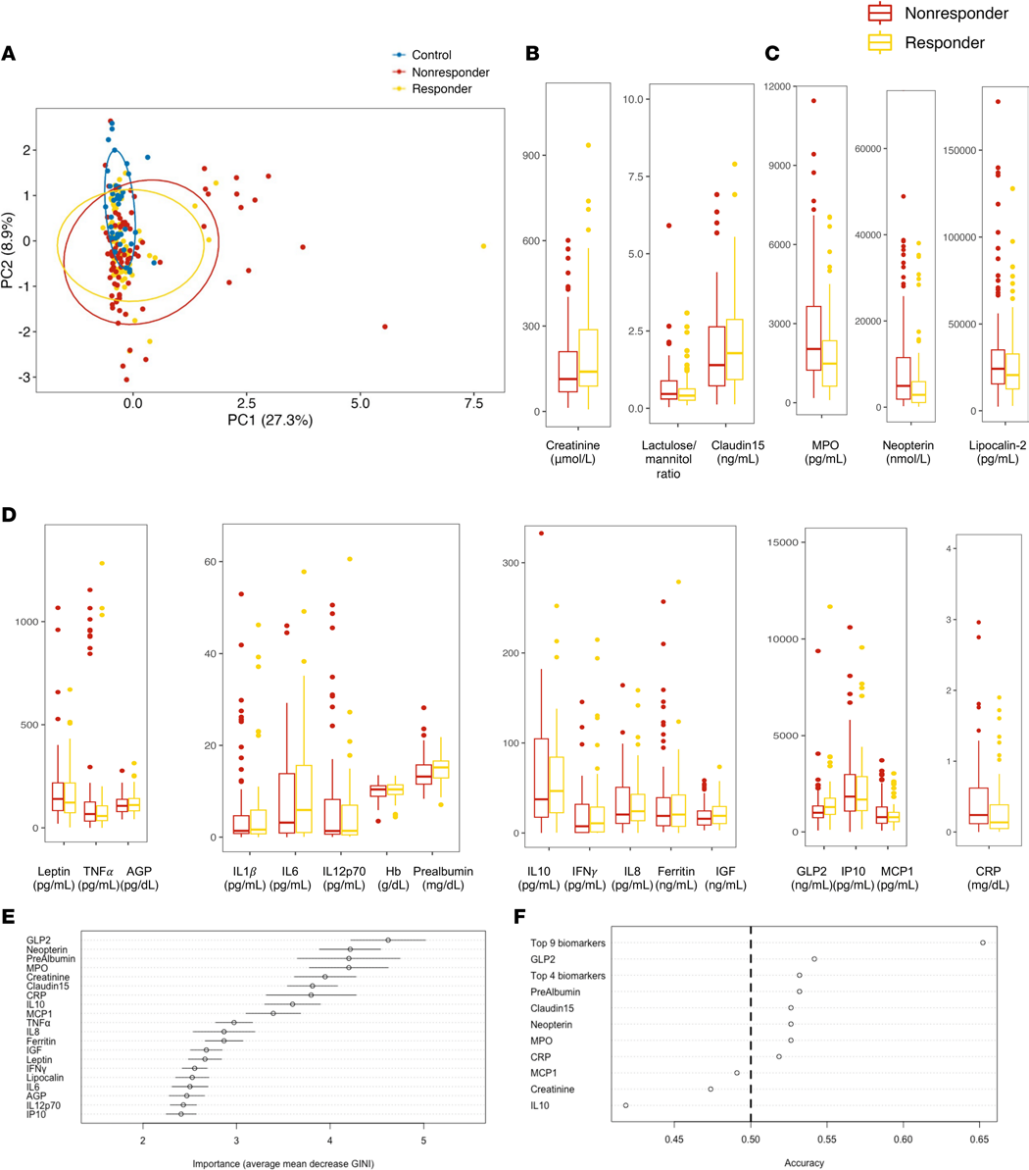
Baseline inflammatory biomarkers predict response to intervention. (**A**) PCA displaying the relationship between biomarkers and cytokines in the responders (*n* = 66), nonresponders (*n* = 82), and controls (*n* = 38) at 9 months of age. (**B**–**D**) Urine (**B**), fecal (**C**), and serum biomarker (**D**) levels from stunted children at 9 months of age prior to RUSF in SEEM cohort. *P < 0.05. (**E**) Variable importance as shown by mean average decrease in GINI for each biomarker in a random forest model trained to predict whether a child would respond to the nutritional intervention. Random forest had a 73% CV accuracy. Circles indicate average importance; data are shown as mean ± SD of 20-fold cross validation. (**F**) Accuracy of logistic regression models trained to predict response from individual biomarkers or combinations of biomarkers deemed important from random forest analysis.

The heterogeneity of biomarker profiles observed in controls suggests a mixed population among this group. Therefore, we hypothesized that biomarkers would help identify which undernourished infants were less likely to respond to RUSF. Relabeling controls by nutritional response (RUSF-R versus RUSF-NR) supported this hypothesis; most samples that failed to cluster with control samples belonged to the nonresponder group (Figure 4A). Heterogeneous samples were more likely to be RUSF-NR; however, RUSF-R and RUSF-NR groups did not cleanly separate, indicating overall broad similarity in biomarker profiles. In univariate analyses, fecal NEO, serum CRP, and IL-10 were higher in RUSF-NR versus RUSF-R before intervention. However, after multiple testing corrections, there were no significant differences observed between controls and controls (Figure 4, B–D). Therefore, we took a machine learning approach to identify biomarkers that are more predictive of RUSF response.

Biomarkers were incorporated into an ensemble of random forest (RF) models with 20-fold cross-validation. Using this approach, biomarker data were able to correctly predict response to nutritional intervention with 60% accuracy (Figure 4E). A distinct set of 9 features (GLP2, MPO, prealbumin, NEO, claudin-15, creatinine, CRP, IL-10, and MCP1) representing a mix of growth and inflammatory biomarkers was identified as most important for distinguishing responders from nonresponders. We observed a trend toward increased markers of growth and nutritional status (GLP2, prealbumin, creatinine) in responders, consistent with the elevation of these markers in control samples relative to controls. We also noted a trend toward increased intestinal inflammation (MPO, NEO) in nonresponders — a difference not seen in comparing controls and controls. IL-10 was significantly higher in responders and CRP was significantly higher in nonresponders, highlighting elevated systemic inflammation as predictive of failure to respond. A logistic regression model was trained based on these 9 biomarkers to interrogate the importance of individual biomarkers. This model correctly predicted response with 68% accuracy on training set data and 73% accuracy on withheld test set data (Figure 4F). Regression models were built using either individual or the collective top 4 biomarkers according to RF performed poorly, with accuracy ranging from 50% to 60%. Broadly, these results indicate that biomarkers of growth, in conjunction with biomarkers of intestinal and systemic inflammation, can reliably distinguish RUSF responders and nonresponders prior to intervention.

### Gut microbiome diversity differs between wasted children and controls.

**Figure 5:**
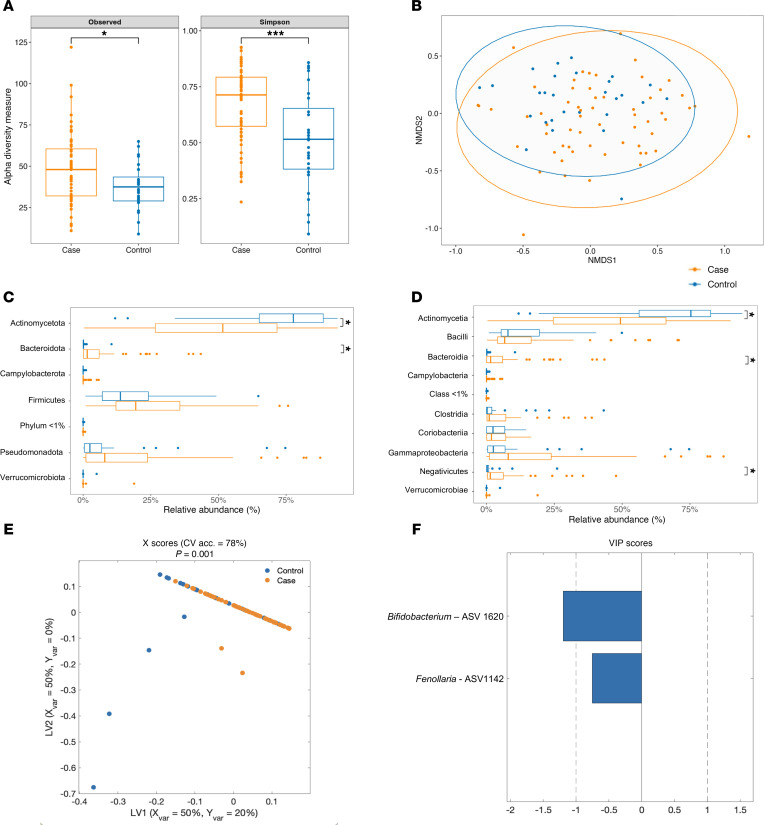
Wasted microbiota are more diverse relative to well-nourished microbiota. (**A**) α Diversity measurements (number of observed species, Simpson index) of wasted controls and healthy controls at 9 months of age prior to RUSF. (**B**) Nonmetric multidimensional scaling plot on Bray-Curtis matrices comparing the community composition of the controls and controls prior to the intervention. (**C** and **D**) Relative abundance of phylum (**C**) and class (**D**) level taxonomy between controls and controls prior to the nutritional intervention. *P < 0.05. An OPLS-DA model was constructed to discriminate between controls and controls using the relative abundance of ASVS from their fecal microbiome. The model outperformed all of 1,000 randomly permuted models (*P* < 0.001). (**E**) Scatter plot of the X scores on latent variables 1 and 2 (LV1 and LV2), where each point represents 1 sample. (**F**) Bar plot shows the variable importance in projection (VIP) scores, artificially oriented in the direction of loadings on LV1, and colored according to their association with patient or control samples, labeled with genus name and ASV number. VIP scores > 1 indicate a variable with greater than average influence on the projection.

Next, we explored the fecal microbiome community profiles of controls (including responders and nonresponders) and controls. We performed 16S rRNA sequencing on stool samples collected at baseline (preintervention) and at several time points after intervention to identify amplicon sequence variants (ASVs), representing unique biological sequences that can be assigned a taxonomy. α Diversity was significantly higher in controls as compared with controls (Figure 5A). This was true when measuring observed ASVs as a measure of richness (how many unique taxa are present) and when measuring Simpson’s diversity, which accounts for both richness and evenness (how evenly the relative abundance of taxa are distributed). Controls and controls also displayed significantly different β diversity, as visualized using nonparametric multidimensional scaling (NMDS) on a Bray-Curtis dissimilarity matrix (Figure 5B). These results suggest that wasted children in our cohort had a distinct microbiome from controls, characterized by increased diversity and greater evenness of taxa.

We next determined which specific phylogenetic groups were different between controls and controls. Consistent with the decreased diversity observed in controls versus controls, the community composition of control samples was largely dominated by Actinomycetota, whereas this phylum made up a significantly smaller proportion of the total community in controls (Figure 5C). The lower relative abundance of Actinomycetota in controls was compensated by a significant increase in Pseudomonadota and trends toward increased Bacillota and Bacteroidota relative to controls. These changes were also reflected at the class level (Figure 5D), with a relative decrease in Actinomycetia in controls and increased Gammaproteobacteria and trends toward increased Bacteroidia, Negativicutes, and Clostridia.

Finally, a LASSO-regularized, orthogonal partial least squares-discriminant analysis (OPLS-DA) model was constructed to identify ASVs that best discriminate between groups (Figure 5E). The model outperformed all 1,000 randomly permuted models (*P* < 0.001) and achieved a cross-validation accuracy of 78% (Supplemental Figure 3A). Variable importance in projection (VIP) scores were plotted for top features and reflected ASV important for discriminating between groups (absolute value of VIP > 1 is an important feature, with the sign of VIP indicating which group it is related to) (Figure 5F). This analysis identified ASV1620, a member of the *Bifidobacterium* genus, as particularly important in discriminating between controls and controls. Univariate analysis showed ASV1620 was significantly enriched in control samples (Supplemental Figure 3B). This ASV is a member of the Actinomycetota phylum and *Bifidobacterium* genus, which are known to be important in early life (9). As LASSO hides features that are linearly correlated to key driver features, ASVs with a high correlation to those identified in the model may also be significant and are shown in a correlation network (Supplemental Figure 3C). Taken together, our results show greater microbiome diversity at the expense of *Bifidobacteria* species is a signature feature of undernourished controls prior to RUSF.

### ASV-level differences reliably distinguish responders from nonresponders among wasted children.

**Figure 6:**
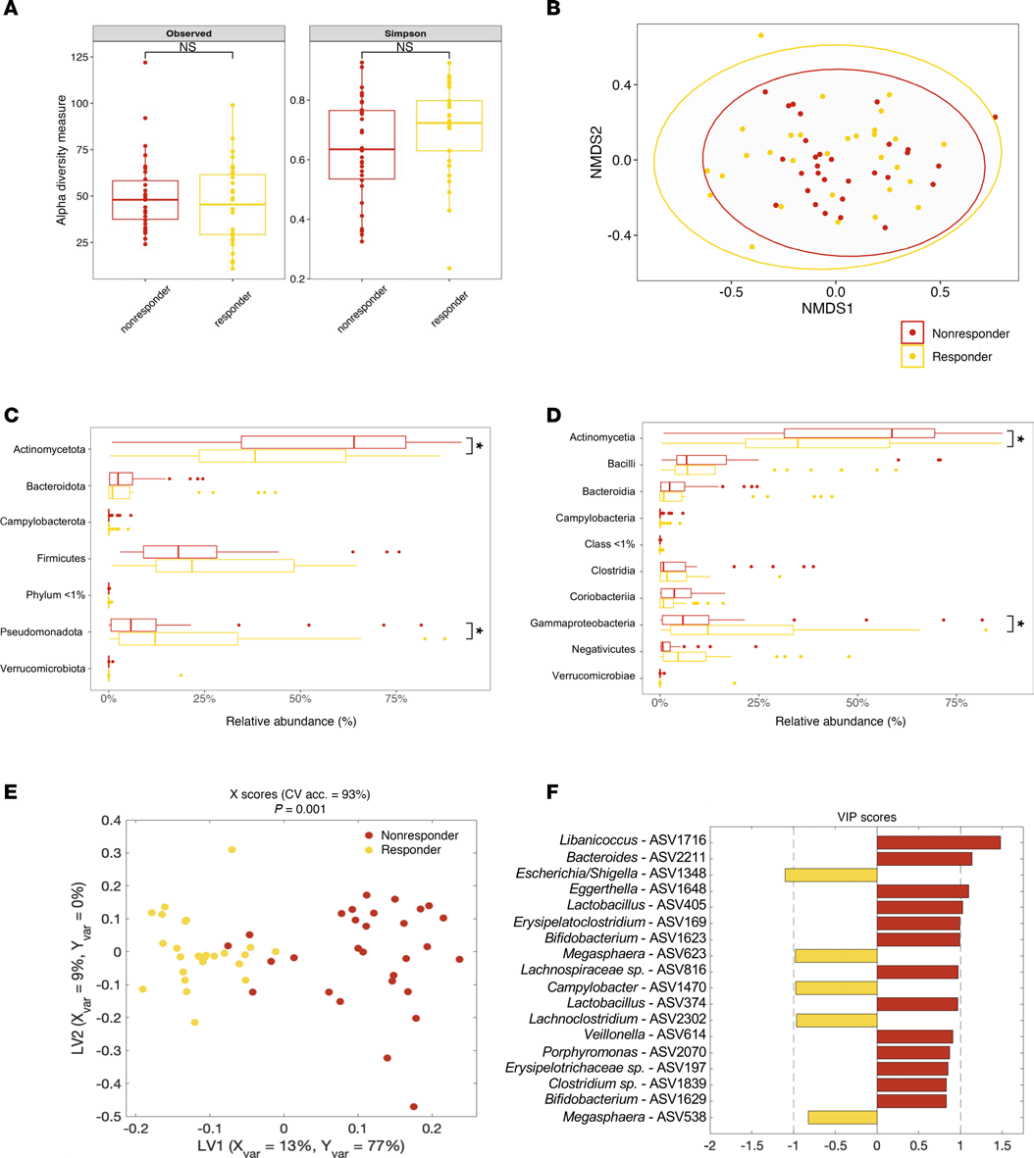
Responders and nonresponders harbor distinct fecal microbiota prior to RUSF. (**A**) α Diversity measurements (number of observed species, Simpson index) of responders and nonresponders at 9 months of age prior to RUSF. (**B**) Nonmetric multidimensional scaling plot on Bray-Curtis matrices comparing the community composition of the responders and nonresponders prior to the intervention. (**C** and **D**) Relative abundance of phylum (**C**) and Class level (**D**) taxonomy between controls and controls prior to the nutritional intervention. An OPSL-DA model was constructed to discriminate between responders and nonresponders using the relative abundance of ASVS from their fecal microbiome. The model outperformed all of 1,000 randomly permuted models (*P* < 0.001). (**E**) Scatter plot of the X scores on latent variables 1 and 2 (LV1 and LV2), where each point represents one sample. (**F**) Bar plot shows the variable importance in projection (VIP) scores, artificially oriented in the direction of loadings on LV1 and colored according to their association with responder or nonresponder samples, labeled with genus name and ASV number. VIP scores > 1 indicate a variable with greater than average influence on the projection.

We next sought to identify compositional differences in gut microbial communities of RUSF responders and nonresponders — both baseline differences and longitudinal changes after intervention. We observed no significant differences in either α or β diversity between responder and nonresponders prior to RUSF (Figure 6, A and B), indicating the gut microbiota of these groups are broadly more similar to one another, relative to more striking diversity differences between controls and controls. Community composition in RUSF-NR mostly consisted of enrichment in Actinomycetota*,* whereas RUSF-R exhibited a significantly lower proportion of Actinomycetota and increased Pseudomonadota (Figure 6, C and D), similar to the differences we observed in controls versus controls. Parallel trends were seen at the class level, where the relative abundance of Actinomycetia and Gammaproteobacteria were significantly higher and lower in RUSF-R, respectively. In addition, we noted trends toward a higher relative baseline abundance of Bacillota at the phylum level and Negativicutes at the class level.

We then generated a LASSO-regularized, OPLS-DA model to distinguish responders from nonresponders based on gut microbiome composition prior to RUSF. Remarkably, this model outperformed all 1,000 randomly permuted models (*P* < 0.001) and achieved a cross-validation accuracy of 93%, indicating distinct differences at the ASV level between RUSF-R and RUSF-NR (Supplemental Figure 4A). VIP scores were plotted and used to determine ASVs important for discriminating between groups (Figure 6, E and F), with univariate analysis showing significant enrichment in several of the important features (Supplemental Figure 4B). ASVs with a high correlation (70%) to those identified in the model that may also be of significance are shown in a heatmap (Supplemental Figure 4C). This analysis points to a distinct pre-RUSF microbiome state characterized by increased colonization of Pseudomonadota in children whose ponderal growth markedly improved after RUSF.

### Responders display shifts in gut microbiome composition during RUSF, and microbiome composition in responders and nonresponders converge in the aftermath of RUSF.

**Figure 7:**
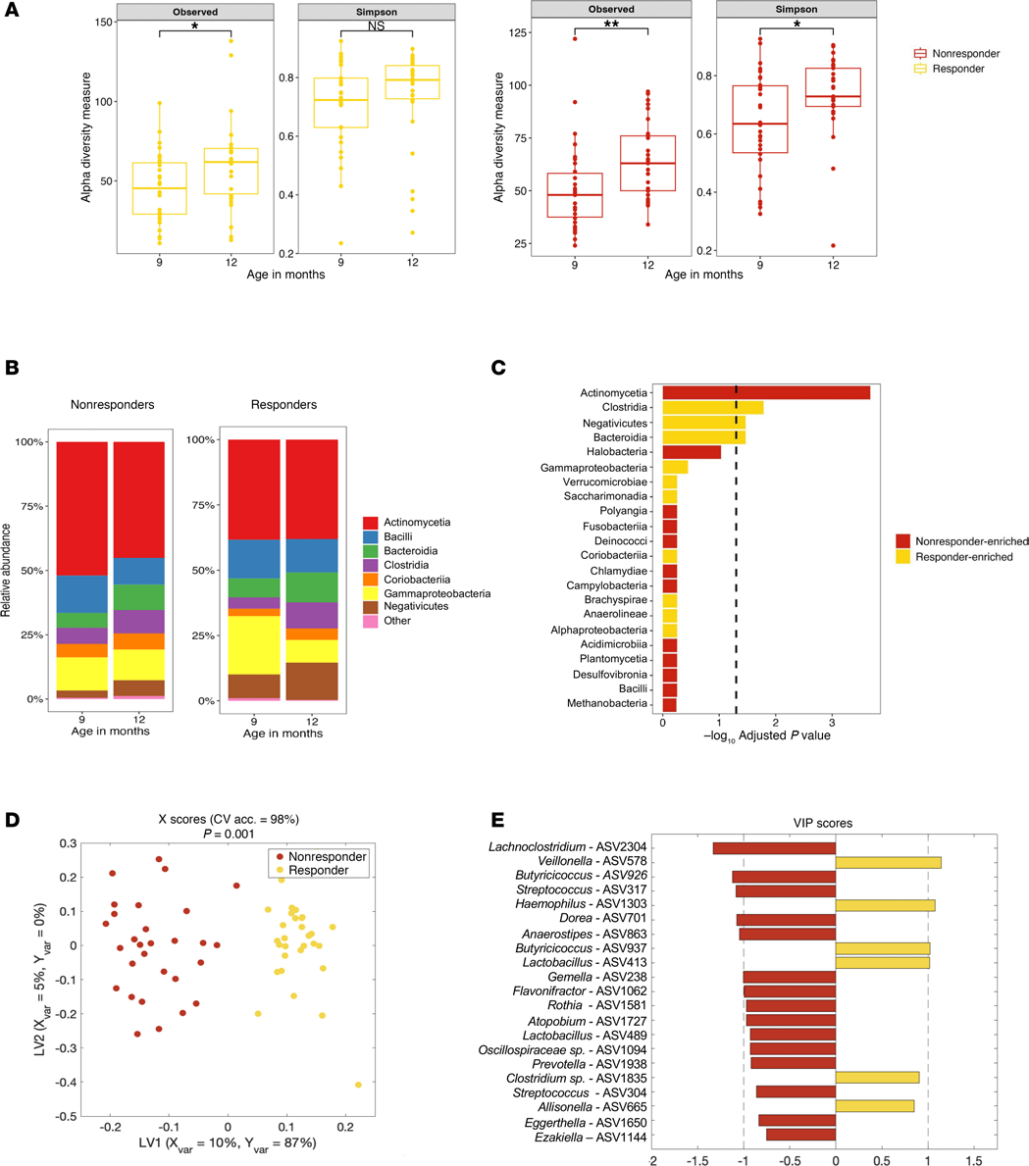
RUSF responders exhibit distinct shifts in gut microbiome composition over the course of the nutritional intervention. (**A**) α Diversity measurements (number of observed species, Simpson index) of responders and nonresponders before and after intervention. (**B**) Comparison of relative abundance of microbial taxa at the phylum level in responder and nonresponder stool samples over the course of the study. (**C**) The –log_10_ adjusted *P* values from a mixed linear effects model comparing the differences in the relative abundance at the class level between responders and nonresponders. The dotted line indicates an adjusted *P* < 0.05. (**D**) Scatter plot of the X scores on latent variables 1 and 2 (LV1 and LV2) from an OPSL-DA model trained to discern differences in ASV relative abundance between responders and nonresponders after intervention, where each point represents 1 sample. (**E**) Bar plot shows the variable importance in projection (VIP) scores, artificially oriented in the direction of loadings on LV1 and colored according to their association with responder or nonresponder samples. VIP scores > 1 indicate a variable with greater than average influence on the projection.

We next turned our attention to longitudinal changes in the microbiome over RUSF supplementation. α Diversity increased during the intervention among responders and nonresponders (Figure 7A), a pattern also observed in controls (Supplemental Figure 5A). We identified no significant shifts in RUSF-NR microbiota at the phylum or class levels. In contrast, in RUSF-R, we identified a significant decline in the relative abundance of Pseudomonadota, largely driven by reductions in Gammaproteobacteria and an increase in Clostridia. This decline in Gammaproteobacteria may explain the ability of responders to gain weight during the intervention as pathogens such as *E*. *coli*, Shigella, and Salmonella are implicated in EED, contributing to malnutrition while disrupting intestinal function. Importantly, we observed a robust increase in the relative abundance of Negativicutes in RUSF-R relative to RUSF-NR (Supplemental Figure 5B) before and after RUSF (Figure 7B and Supplemental Figure 7C). In RUSF-NR, taxa were not immediately different before versus after intervention. At the family level, shifts in the Negativicutes class were driven by members of *Veillonellaceae*, Pseudomonadota by members of *Enterobacteriaceae,* and Actinomycetia by members of the *Bifidobacteriaceae* family (Figure 7C).

To identify specific ASVs associated with RUSF response, we constructed a LASSO regularized OPLS-DA model to discriminate between RUSF-R and RUSF-NR samples immediately after intervention (12 months of age) (Figure 7D). The model outperformed all of 1,000 randomly permuted models (*P* < 0.001) and achieved a cross-validation accuracy of 98% (Supplemental Figure 6A). A plot of VIP scores showed several ASVs as important in discriminating between responder and nonresponder samples after intervention. ASV2304, a member of the *Lachnoclostridium* genus, was the most important feature of RUSF-NR samples (Figure 7E). Univariate analysis showed this ASV to be present significantly more often in nonresponder samples than in responder samples. The most important microbial feature relevant to RUSF-R samples was ASV578, a member of the *Veillonella* genus and Negativicutes class that was present more abundantly in responders (Supplemental Figure 6B). ASVs with a high correlation (>70%) to those identified in the model that may also be of significance are shown in a heatmap (Supplemental Figure 6C). Together, this analysis shows a RUSF-driven transformation of the gut microbiome in RUSF-R distinct from that observed in RUSF-NR over the course of the nutritional intervention. This transformation is characterized by a robust decrease in Gammaproteobacteria and an increase in Negativicutes.

### Systemic inflammation identifies RUSF-NR outliers with responder-like microbiome profiles.

**Figure 8:**
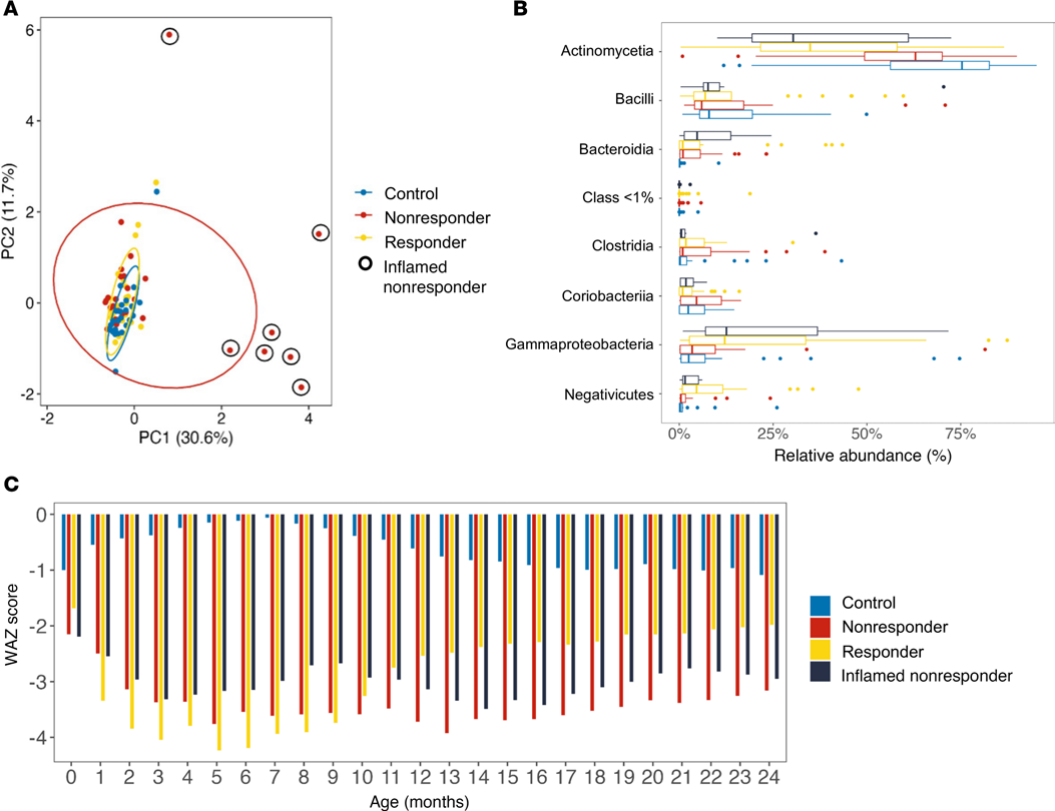
RUSF nonresponders with high levels of inflammation harbor fecal microbiota that resemble those of responders. (**A**) PCA displaying the relationship between biomarkers and cytokines measured before intervention from patients with matching microbiome data in the responders (*n* = 30), nonresponders (*n* = 30), and control groups (*n* = 28). Black circles denote nonresponder samples with high levels of inflammation selected for downstream analysis. (**B**) Comparison of the average relative abundance of microbial taxa at the phylum level in responder, nonresponder, and inflamed nonresponder stool samples prior to the nutritional intervention. (**C**) Longitudinal change in WAZ score for control, responder, nonresponder, and inflamed nonresponder children over the first 2 years of life.

Among the 30 worst RUSF-NR, we identified 7 individual nonresponders with distinct biomarker profiles relative to either controls or RUSF-R. These profiles were primarily driven by a significant elevation in biomarkers of systemic inflammation (Figure 8A). We next asked whether these individuals harbored distinct microbiota relative to other RUSF-NR. The microbiomes of inflamed RUSF-NR showed greater similarity to RUSF-R than to noninflamed RUSF-NR (Figure 8B). This was most evidenced by lower levels of Actinomycetota and increased levels of Pseudomonadota in inflamed versus noninflamed RUSF-NR. Inflamed RUSF-NR also exhibited distinct growth trajectories, with higher WAZ scores prior to RUSF followed by marked decreases in WAZ during the intervention (Figure 8C). Follow-up analysis also showed that these infants experienced higher rates of diarrheal episodes and acute respiratory infections (Supplemental Figure 7, A and B, Supplemental Table 3). These findings suggest that microbiome structure and function play an important role in RUSF response; however, systemic inflammation and acute illnesses can undermine that response.

### Validation of the predictive model.

After pinpointing microbial taxa with strong predictive potential, we validated our findings in an independent Bangladeshi cohort of young children. Since limited studies were suitable for validation, we selected the 2022 study by Chen et al. (13) due to its utilization of a chickpea-based MDCF. We utilized the data of 60 children who received the MDCF and categorized them into 3 groups based on changes in WAZ during the intervention (Supplemental Figure 8A). The fecal microbiome was compared between children showing the most favorable response and those showing the least. Unlike our dataset, at the preintervention stage, significant differences in Pseudomonadota or Negativicutes abundance in the Chen et al.(13) study were not seen (Supplemental Figure 8B). To uncover any consistent microbial taxa with predictive potential across cohorts, we first used PCA on preintervention composition data (Supplemental Figure 8C) and, seeing no significant batch effects, trained an Orthogonal Partial Least Squares Discriminant Analysis (OPLS-DA) model using preintervention microbiome data from the SEEM study and applied it to the preintervention validation dataset (Supplemental Figure 8D). Although our model identified several taxa significantly linked to response, it only classified patients with 54% accuracy in the validation dataset. While this accuracy is only marginally better than chance, it underscores the microbiome composition variations between cohorts and studies.

## Discussion

We identified host-microbiome determinants of RUSF efficacy among a birth cohort of rural Pakistani infants at high risk for moderate and severe acute malnutrition. Through a comprehensive assessment of (a) baseline biomarkers of EED and systemic inflammation, (b) the structure and flux of gut microbial communities before and after RUSF, and (c) ponderal growth responses to RUSF, we uncovered several findings with important implications for the evaluation and treatment of acute childhood undernutrition in LMIC community settings. First, pre-RUSF biomarker profiles reliably distinguished RUSF responders from nonresponders; children with heightened gut and systemic inflammation were less likely to respond to supplementation. Second, we identified key baseline differences in the gut microbial community structure of nourished infants, RUSF responders, and RUSF nonresponders. Third, in contrast to nonresponders, RUSF responders displayed significant shifts in gut microbiota over the course of supplementation. Fourth, in a small group of outliers whose gut microbiome would have otherwise predicted positive RUSF response, high burdens of inflammation and infections undermined RUSF efficacy. Lastly, the predictive power of these and other measurable host-microbiome factors sets the stage for precision nutrition approaches to not only tailor the formulation of RUSF — or microbiome-directed complementary foods — to achieve greater efficacy but also shape the timing, duration, and adjunctive therapies to RUSF to promote optimal and durable growth outcomes.

RUSF responders weighed more during the first month of their life, further supporting the role of antenatal care and birth dynamics in establishing childhood growth trajectories (14). Univariate comparisons of biomarkers revealed increased intestinal inflammation in RUSF-NR and increased markers of muscle mass (urinary creatinine) and the antiinflammatory cytokine IL-10 in responders. Our models identified a set of 9 biomarkers with acceptable predictive accuracy for children at risk of RUSF nonresponse. These included established biomarkers for growth (prealbumin, creatinine, leptin, GLP-2, and IGF-1) as well as commonly measured markers of gut and systemic inflammation (fecal MPO, serum AGP, and CRP). These biomarkers were tested because of the known microbial infections in environmental enteropathy (15, 16). Together, increased growth biomarkers and antiinflammatory cytokines may indicate that RUSF-R was primed to break out of the vicious cycle of inflammation and undernutrition once supplementation began. Our analysis provides valuable insights into the role of baseline biomarkers in identifying those who may require longer or additional therapeutic interventions in community settings.

RUSF responders and nonresponders displayed significant differences in microbiome composition before intervention. Although diversity indices were similar, responders harbored higher levels of Gammaproteobacteria and Negativicutes and lower levels of Actinomycetia prior to RUSF. A PLS-DA model trained to predict who would respond to RUSF based on the relative abundance of preintervention ASVs was able to classify responders with 93% accuracy, identifying several ASVs correlated with response versus nonresponse. Another key observation was that nonresponders exhibited minimal changes in gut microbiome composition, whereas responders displayed a high degree of microbiome restructuring over supplementation. Gammaproteobacteria, a large class of bacteria that includes the pathogens such as *Klebsiella*, *Salmonella*, and *Shigella*, were found in higher numbers before the intervention and decreased significantly thereafter in responders. The high prevalence of these facultative anaerobes prior to intervention may have contributed to earlier growth faltering in these children. RUSF appeared to sufficiently restructure the microbiome of responders toward growth-conferring taxa, with significant increases in Negativicutes, including *Megasphera* and *Veillonella*. Recent studies show these taxa ferment chickpeas to release beneficial metabolites (17), which might account for their increased presence in Acha Mum responders.

In concordance with prior observations that inflammation and repeated infections abrogate catch-up growth in childhood (18, 19), we found that nonresponders with the highest burden of inflammation, diarrhea, and respiratory infections harbored microbiomes more similar to responders rather than typical RUSF nonresponders. This may indicate that this small group of infants was primed to respond to RUSF but derailed by acute illness and inflammation. Hence, gut and systemic inflammation and the state of the gut microbiome may jointly coordinate the metabolic response to RUSF. Future studies should explore targeting the reduction of acute inflammation and/or infection before or during the administration of RUSF as a strategy to increase RUSF efficacy (20, 21).

We attempted to validate the findings of this study in another cohort of malnourished children. While the preintervention time point data could accurately predict response in this dataset, the key taxa that distinguished responders and nonresponders differed from our cohort. While the Chen et al. (13) dataset best matched our study and used a chickpea-based intervention, their study design had several key differences. First, while our study occurred in an arid rural region in Pakistan, the validation study occurred in Dhaka’s humid urban environment. Second, while our study included children with both SAM and MAM, the validation study included only children with MAM. Finally, the intervention began at 9 months in our study, whereas the validation study occurred between the twelfth and eighteenth months of life. Together, these factors may all contribute to the different trends observed in microbiome restructuring.

This study benefited from several strengths. First, we included well-nourished controls from the same rural settings who did not require RUSF, providing a set of local, age-matched controls. Second, we tested various blood, stool, and urine biomarkers to distinguish relative contributions from EED versus systemic inflammation. Third, the longitudinal collection of fecal samples enabled us to observe the restructuring of the microbiome and correlate this restructuring with the RUSF response. Lastly, by monitoring anthropometrics up to 24 months of age, we explored the intervention’s long-term effects and identified delayed onset improvements in ponderal growth among RUSF nonresponders. The limitations of our study include performing serial 16S rRNA fecal analyses on only 28–30 individuals per group. Our focus on extreme RUSF responders and nonresponders to interrogate WAZ-associated microbial taxa may have missed more subtle host-microbiome interaction in children who showed a mild benefit from the intervention. We did not collect fecal samples during the intervention, which might have better captured the dynamics of microbiome transformation. Since we did not quantify these pathogens using PCR approaches and with the limited sequencing depth provided by 16S rRNA analysis, confirming any potential probiotic/symbiotic candidates in gnotobiotic animal models or human nonclinical models — such as EED-on-a-chip — is critical in translating these findings into an intervention study (22) and would be important before clinical interventions. Although machine learning and other computational tools allowed us to identify important ASVs, the heterogeneous nature of gut microbiota means important features should be interpreted with caution. Lastly, among the wasted children, clinical data on oedematous malnutrition was not available, nor was an independent biomarker of compliance assessed; instead, we reported it based on the caregiver’s information.

For future studies of RUSF, we recommend exploring longer durations of intervention, especially in primary nonresponders — a group in whom we observed later convergence in the transformation of several ASVs. In responders, growth improvements with RUSF were durable; however, continued monitoring and support during critical growth periods is warranted. The correlations we identified between birthweight and RUSF response at 9 months of age underscore the importance of healthy pregnancy and birth anthropometrics in establishing growth trajectories. Our finding that common illnesses and inflammation undermined RUSF response reemphasizes the importance of clean water, sanitation, and hygiene practices and access to essential drugs in optimizing the individual and community effect of RUSF. Adjunct therapy with probiotics tailored to RUSF or MDCF composition and the predominant infant gut microbial signatures of at-risk populations may enhance outcomes and is an ongoing area of study (23). We suggest that future studies aimed at identifying bacteria associated with response to a nutritional intervention be designed to include multiple sites to better account for and understand how cohort setting affects response. Finally, for children with either microbiome or inflammatory states associated with nonresponse, administering an antibiotic or antiinflammatory medication prior to RUSF might conceivably prime the microbiome and gut for a more beneficial ponderal growth response.

We conclude that birth weight, gut and systemic inflammation, and gut microbial community composition profoundly influence the ponderal growth response of acutely undernourished infants to RUSF. The transformation of gut microbial community structure over RUSF supplementation further shapes this response. Multicentered studies designed to validate promising biomarkers, microbial targets, and RUSF formulations — using harmonized methods across LMIC settings where the burden of acute and chronic childhood undernutrition is greatest — are urgently needed to develop scalable solutions toward global reductions in childhood wasting, underweight, and stunting.

## Methods

### Sex as biological variable

This study involved both the sexes, as we enrolled 38 males and 22 female infants. While investigating demographic, biomarker profiles, and microbiome signatures, similar findings are reported for both sexes.

### Study participant details

Study participants were drawn from the SEEM-Pakistan birth cohort study (*n* = 416) conducted in Matiari, Sindh, Pakistan. In this community-based intervention study, children were longitudinally followed from birth to 24 months of age (24). Controls and controls were enrolled from the same population (rural setting). Blood, urine, and fecal samples were evaluated for potential EED biomarkers. The controls received appropriate educational and nutritional interventions. At the same time, selected nonresponders underwent an evaluation to determine eligibility for further workup, including upper gastrointestinal endoscopy for histopathological workup, characterization of mucosal gene expression, and exploration of duodenal microbial community composition (25). The SEEM study is part of a global effort to establish histopathological findings from children at risk of developing EED and to identify mechanism-based biomarkers (26, 27). For enrollment in the SEEM study, newborns (0–1 months) were screened at 0–3 months of age, and followed monthly for growth monitoring. Based on 2 consecutive months of anthropometrics, 3- to 6-month-old infants were enrolled either as controls (WLZ ≥ 0, LAZ ≥ –1) or wasted controls (WLZ ≤ –2). Out of 350 controls, 187 were selected for RUSF nutritional intervention after not responding to a caregiver nutrition education program. Response to the Acha Mum RUSF intervention was monitored as improvement in WLZ ≥ –2 one week after postintervention, irrespective of their WLZ at the preintervention timepoint. In total, 41% of controls responded in the SEEM study. For this study, we selected participants based on the availability of longitudinally collected fecal samples. Of that 41% of individuals who responded to the intervention in the parent SEEM study, we selected 30 children with the highest level of response, a maximum gain in WAZ (change > 0.5), and classified them as RUSF-R while from those 59% nonresponders; we selected the children (*n* = 30) who experienced a decline in WAZ or no change and labeled them as RUSF-NR. The fecal samples of these 30 best responders and 30 worst nonresponders based on the change in WAZ were selected for the 16S analysis in this substudy

### Data collection

Information on general demographic characteristics was collected by trained community health workers through interviews with mothers, which included children’s birth data, breastfeeding history, and parental parameters. Anthropometric measurements were performed using standard procedure and equipment, with weight measured to the nearest 20 g precision electronic scale (TANITA 1584) and length to the nearest 1 mm using a rigid length board with a movable footpiece. These measurements were collected monthly for up to 24 months and converted to *z* scores using the WHO Anthro 3.2 application. Based on established cut-offs for nutritional indicators, the participants were categorized as stunted (LAZ < –2), underweight (WAZ < –2), at risk of wasting (WLZ < –1 and ≥ –2), moderately wasted (WLZ < –2), and severely wasted (WLZ < –3), indicating levels of nourishment.

### Sample collection for inflammatory biomarkers

Blood, fecal, and urine samples were collected from participants at 9 months of age (Figure 1B). In total, 1–2 mL of blood was collected from which serum was aliquoted in small volumes to avoid freeze-thawing and transported at 4°C from the field site lab to the Infectious Disease Research Laboratory (IDRL) where they were stored at –80°C until processed. Commercial ELISA kits were used for estimation of GLP-2 (USCN, Life Sciences Inc.) while CRP, ferritin, and AGP were analyzed using a Hitachi 902 analyzer (Roche Diagnostics), and IGF-1 was measured using a LIAISON (Diasorin Saluggia). All assays were performed following manufacturer protocols. For fecal samples, the child’s caretaker collected the sample using a wooden spatula and put it into a clean container provided in the stool collection kit. The fecal sample was transferred into multiple cryogenic vials for long-term storage. Commercial ELISA kits were used for the estimation of MPO (Immunodiagnostic AG, Stubenwald-Allee, and Bensheim) and NEO (GenWay Biotech). Fecal LCN2 was measured by DuoSet ELISA DY1757. All plates were read on a Biorad iMark plate reader.

For the evaluation of serum cytokines, a commercially available MILLIPLEX MAP Human Cytokine/Chemokine (MERCK) kit was used. The screening panel including IFN-γ, IL-10, IL-12 (IL-12p70), IL-1β, IL-6, IL-8, IP-10 (also called as CXCL10), MCP-1 (also called as CCL2) and TNF-α as per manufacturer’s instructions using Bioplex-200 instrument. The data were analyzed using Bioplex Manager 6.1.

### Administration of nutritional intervention

Enrolled controls with WLZ < –2 at 9 months were selected for nutritional intervention. Acha Mum, a RUSF, was given at a dose of 1 sachet per day to controls with MAM (WLZ scores between –2 and –3), while children with SAM (WLZ < –3) were administered sachets as per child’s weight (200 kcal/kg/day) (28). Acha Mum’s composition is provided in Supplemental Table 4. This 8-week intervention was monitored through weekly visits by the local team to document compliance, side effects, needs for medical assistance, and other details. Compliance was calculated (weekly) based on the empty wrappers returned by mothers (Compliance = [total packet used/total packet given] × 100). One week after the completion of the intervention, the response was measured as the overall change in the WAZ of the children.

### Longitudinal fecal sample collection for 16S ribosomal RNA gene sequencing

Mothers of study participants were trained to collect the fecal samples and were provided with mobile cards for timely communication with the collection team in the field. As described above, the mothers collected the fecal sample and sent an immediate message to the field team to pick up the sample within 30 minutes. Overall, 4 samples were collected from each child: 1 sample at 9 months to capture microbiota at the preintervention stage, while 3 samples were collected at 12, 13, and 18 months to evaluate postintervention taxa (Figure 1B). Fresh samples were transferred into a prechilled cryovial, snapped to the aluminum cryo cone and placed into a freezing container (Coleman) at 2°C–8°C. The time taken from the passage of fresh stools to being snap-frozen in a dry shipper was less than 30 minutes. The Coleman was carried in a liquid nitrogen dry shipper to the local laboratory and later shifted to the IDRL at Aga Khan University, on dry ice, where samples were stored at –80°C until shipped to BGI Genomics (formerly Beijing Genomics Institute). A 30 ng qualified DNA template was tested for sample integrity by agarose gel electrophoresis and concentration by a microplate reader (Qubit fluorometer). The quantified samples (6–100 ng/μL) were normalized to 30 ng DNA per reaction. Fusion primers were designed to include Illumina adapter sequences, an 8-nucleotide index sequence, and a gene-specific primer and were added to the PCR reaction system. All PCR products were purified by Agencourt AMPure XP beads, dissolved in an Elution Buffer, and labeled for library construction. Library size and concentration were detected by Agilent 2100 Bioanalyzer. Qualified libraries were sequenced pair-end on the HiSeq 2500 platform according to their insert size. The raw reads were filtered to remove the adapter and low-quality bases. Paired-end reads were added to the tags by Fast Length Adjustment of the SHort read program (FLASH, v12.11). The sequence data are deposited in the NCBI Sequence Read Archive (SRA) under accession no. PRJNA1268017. α Rarefaction curves were generated to assess the effect of sampling depth on ASV abundance (Supplemental Figure 9). The plots indicate that the detection of ASVs had already attained a plateau of 60,000 reads. This trend was separately confirmed in the 3 groups.

### Statistics

#### 16S ribosomal RNA gene analysis and statistical analysis.

Sequence analysis was performed in R using DADA2 (version 1.22.0) (29). The forward read was truncated to 200 bp and reads with ambiguous N bases and > 2 expected errors were removed. Chimeras were removed. Forward and reverse reads were aligned, resulting sequence variant (SV) counts and taxonomic calls were assigned using the SILVA 138 SSU Ref NR 99 (https://www.arb-silva.de/documentation/release-138). Models were created, and data were analyzed in R using Phyloseq (v. 1.38.0), caret (v. 6.0.92), GGplot2 (v. 3.3.6), mixOmics (6.18.1), and nlme (3.1) and in python using Jupyter notebooks, pandas, scipy, numpy, matplotlib, and seaborn. Venn diagrams were created using the R packages VennDiagram (1.7.3) and ggvenn (0.1.9). Radar plots were created using the R package fsmb (0.7.3). Wilcoxon rank-sum tests were used to compare the α diversity and genus relative abundances and numeric anthropometric measurements while χ^2^ tests were used to compare categorical anthropometric measurements. PERMANOVA tests were used to determine statistical significance for β-diversity. To calculate statistics, either the Python package Scipy.Stats package or R was used.

#### Model generation.

Cytokines with several missing measurements across patients were excluded, and patients with missing multiple cytokine measurements were excluded, leaving a dataset with no missing values consisting of 148 observations (children) and 23 variables (biomarkers listed in Supplemental Table 2). An ensemble-based approach was used to identify the cytokines with the highest predictive power in distinguishing responders from nonresponders. In each iteration, a RF model was trained on a randomly sampled 95% of patients. This process was repeated 20 times, such that each patient was left out once, and out-of-bag error rates and variable importance scores were recorded for each iteration. The mean ± SD of variable importance scores across all models were computed and plotted to identify the most predictive cytokines. To assess the predictive power of the selected cytokines, responders and nonresponders were separately split into training and test sets at a 70:30 ratio before being recombined. Logistic regression models were then trained using individual cytokines and groups of cytokines on the training set and evaluated on the test set. The R package randomForest (4.7-1.1) was used to generate a RF model to classify the samples according to the response to the nutritional intervention and determine important features. The forest used has 1,000 trees with a node split (mtry) set to the default of the square root of the number of samples. Mixed linear effects models were used to account for repeated sampling from individual patients when comparing changes in microbiome composition.

OPLS-DA models were created using MATLAB using a custom pipeline initially developed by Remziye Wessel. ASV counts were used as inputs to the models. LASSO regularization is used better to guide the feature selection and model fitting process and improves classification by allowing the selection of a subset of the covariates instead of using all of them. Here, a 5-fold cross-validation was repeated 1,000 times to calibrate the model’s performance. Permutation testing results shuffling the labels of the samples show the goodness of fit of the model versus a null distribution, and the cross-validation accuracy showing the stability of the model are reported in Supplemental Figures S3, S4 and S6. PCA was performed in R and plotted using ggbiplot (0.55). Correlations between SV abundance and biomarker levels were plotted using corrplot (0.92).

#### Validation modeling.

As described above, raw sequencing data from Chen et al. (13) was processed in R according to the DADA2 pipeline. Change in WAZ was calculated by comparing the preintervention score (day 0 in the original paper) and after intervention (day 90 in the original paper). Children were ranked according to the change in WAZ, and the best 20 and worst 20 responders were compared. Data were transformed into percent composition at the phylum, class, and family levels. These data were imported into Python and combined to form a matrix with percent composition data across the 3 taxonomic levels, and this process was repeated for the SEEM data. PCA and PLS modeling were performed using the sklearn package in Python (30).

### Study approval

The study was approved by the Aga Khan University Ethical Review Committee (ERC no. 2021-0535-19973), and written informed consent was obtained from the participant’s parents. The study was registered at ClinicalTrials.gov ID NCT03588013.

### Data availability

All code is available on GitHub (https://github.com/gabehanson/SEEM_microbiome_analysis; commit ID 8f9e34b). All data points for figures with means are included in the Supporting Data Values file. Raw and additional data available upon request.

## Author contributions

SRM, SAA, ZJ, and JI were involved in conceptualization of this study. ZJ, GFH, GBM, JI, and NTI contributed to the methodology. SA, FU, AH, FK, KA, KS, and FA contributed to the investigation of the study. GFH and ZJ led the visualization of the data. SAA and SRM were responsible for the funding acquisition. Project administration included NTI, SA, FU, and AH. SRM and SAA supervised the study. ZJ and GFH were involved in writing the original draft, while SRM, GBM, IM, JZM, JI, NTI, and SAA reviewed and edited the manuscript. ZJ and GFH share co–first authorship, as ZJ conceived the study design around the hypotheses and generated all primary data. GFH led the data analysis, developed the machine learning strategy, and directed its implementation.

## Supplementary Material

Supplemental data

ICMJE disclosure forms

Supporting data values

## Figures and Tables

**Table 1:**
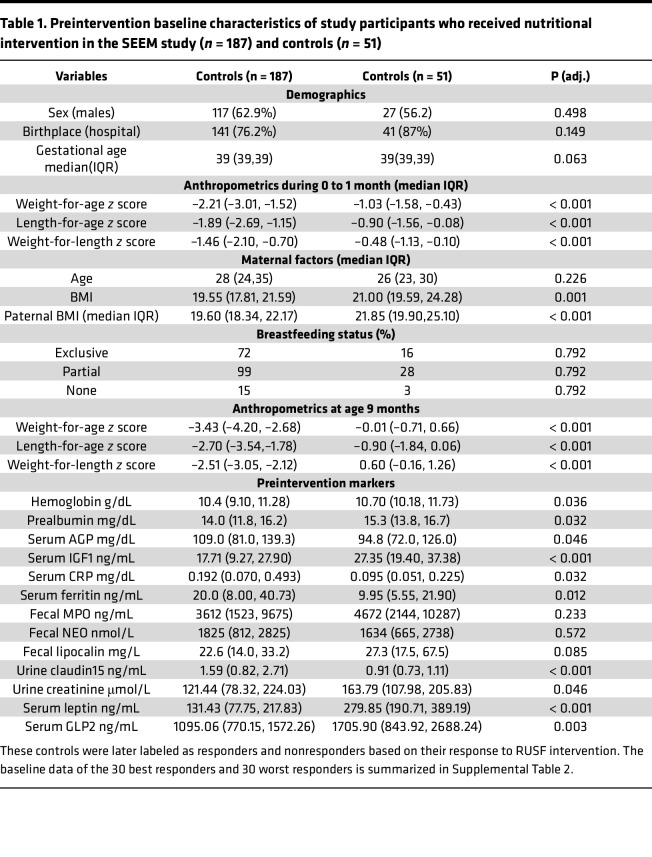
Preintervention baseline characteristics of study participants who received nutritional intervention in the SEEM study (*n* = 187) and controls (*n* = 51)

**Table 2:**
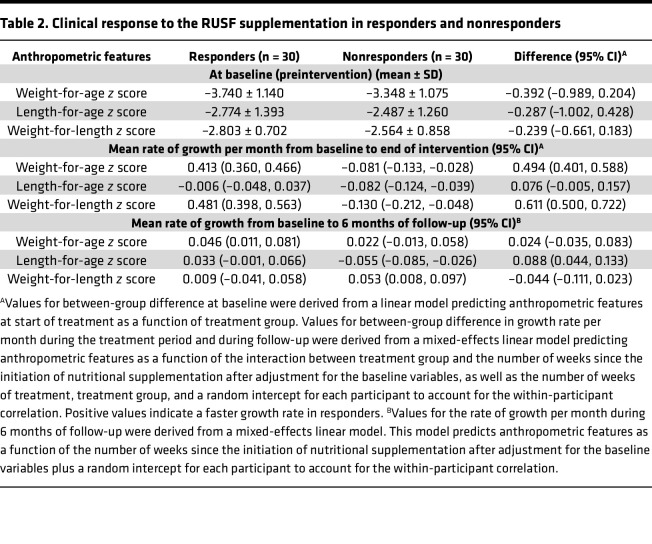
Clinical response to the RUSF supplementation in responders and nonresponders

## Version 1 07/22/2025

Electronic publication
