# Supplementary material for: Host-microbiome determinants of ready-to-use supplemental food efficacy in acute childhood malnutrition
Source: JCI Insight. 2025 Jul 22;10(14):e188993. doi: 10.1172/jci.insight.188993 (PMC12288982; doi:10.1172/jci.insight.188993)
Supplement: Supplemental data [file jciinsight-10-188993-s258.pdf]

Supplementary Results

Baseline clinical characteristics for case (n=60) and control (n=28) infants for whom complete biomarker and fecal 16S microbiome data were available are summarized in **S Table 1**. Maternal and paternal BMI as well as birth anthropometrics were significantly lower in cases vs. controls (P <0.05), differences that became even more pronounced by nine months of age (P <0.05). Breastfeeding patterns were similar between groups over the first six months of life. Cases in which complete biomarker data were available broadly represented the larger SEEM cohort, as shown in **S Table 2**. The comparison between the baseline characteristics of the larger SEEM cohort and those selected for 16S fecal microbiome analysis is summarized in S Table 3, reporting these selected cases and controls as true representatives of their respective larger groups.

S Table 1: Comparison of baseline characteristics between those cases with 16S fecal microbiome analysis (responders and nonresponders combined) included in this sub-study and the selected controls

Variables	Wasted cases (n=60) (32%)	Controls (n=28) 54%	P
Demographics			
Gender (males)	39 (65%)	18 (64.3%)	0.940
Birthplace (hospital)	43 (71.6%)	24 (85.7%)	0.120
Gestational age median(IQR)	39 (38, 39)	39 (39, 39)	0.054
Anthropometrics during 0 to 1 month (median IQR)			
Weight-for-age z score	-1.99 (-2.69, -1.32)	-1.03 (-1.39, -0.31)	< 0.001
Length-for-age age z score	-1.59 (-2.51, -1.06)	-0.88 (-1.54, -0.16)	< 0.001
Weight-for-length z score	-1.39 (-1.99, -0.72)	-0.45 (-1.16, -0.09)	< 0.001
Maternal factors (median IQR)			
Age	27 (23.5, 35)	27 (25, 30)	0.263
Body mass index	19.44 (17.63, 21.89)	20.92 (19.76, 24.46)	0.045
Paternal BMI (median IQR)	19.39 (18.47, 21.89)	22.46 (20.27, 24.32)	0.012
Breastfeeding status (%)			
Exclusive	22 (36.7%)	10 (35.7%)	0.238
Partial	33 (55%)	15 (53.6%)	0.238
None	5 (8.3%)	1 (3.3%)	0.238
Anthropometrics at age 9 months			
Weight-for-age z score	-3.47 (-4.10, -2.67)	-0.11 (-0.72, 0.27)	<0.001
Length-for-age age z score	-2.59 (3.55, -1.75)	-0.88 (-1.88, -0.06)	<0.001
Weight-for-length z score	-2.56 (-3.09, -2.12)	0.53 (-0.17, 1.12)	<0.001
Pre-intervention markers			
Hemoglobin	10.45 (9.2, 11.4)	10.45 (9.5, 11.3)	0.267
Pre-albumin	14.2 (11.55, 16.15)	15.2 (13.6, 16.35)	0.322
Serum AGP mg/dl	116.445 (91, 138.05)	115 (85, 134)	0.207
Serum IGF1 ng/ml	17.67 (10.41, 31.73)	24.46 (17.57, 36.48)	0.055
Serum CRP	0.202 (0.061, 0.56)	0.1295 (0.066, 0.368)	0.030
Serum Ferritin	21.8 (11, 55)	16.95 (5.8, 34.8)	0.212
Fecal MPO ng/ml	3550 (1575, 8829.75)	4672.25 (2640.25, 9275)	0.396
Fecal NEO nmol/L	1925 (1042.15, 2875)	1341.5 (485.95, 2600)	0.914
Fecal Lipocalin mg/L	27.42 (16.75, 41.39)	40.09 (20.19, 94.75)	0.024
Urine claudin15	2.06 (0.85, 2.87)	0.924 (0.69, 1.15)	0.000
Urine creatinine µmol/L	125.63 (91.94, 263.56)	176.57 (129.65, 212.41)	0.841
Serum Leptin ng/ml	145.06(94.95, 260.196)	258.17 (179.39, 345.51)	0.071
Serum GLP2	1156.46 (863.86, 1532.66)	1260.87 (649.51, 2286.14)	0.597

Supplementary Table 2: Baseline characteristics compared between cases only (30 best and 30 worst responders).

Variables	Wasted children (16S)<br>Responder (n=30)<br>Median (IQR)	 nonresponder (n=30)<br>Median (IQR)	p-values
**Demographics**			
Gender (male)	19 (63.4%)	20 (66.7%)	0.791
Birthplace (hospital)	22 (73.4%)	21 (70%)	0.779
Gestational age (weeks)	39 (38, 39)	39 (39, 39)	0.683
**Birth anthropometrics**			
WAZ	-1.65 (-2.34, -0.96)	-2.09 (-2.69, -1.74)	0.980
LAZ	-1.51 (-2.14, -0.93)	-1.77 (-2.83, -1.07)	0.447
WLZ	-1.02 (-1.81, -0.32)	-1.86 (-2.12, -1.17)	0.041
**Maternal factors**			
age at time of delivery, years	30 (24, 38)	26 (23, 35)	0.582
weight at enrollment, kg	47 (42.5, 54)	45 (40, 48.5)	0.448
Height (cm)	154.09 (151, 156.59)	152.19 (149.5, 156)	0.171
BMI	19.98 (17.97, 22.04)	18.69 (17.15, 21.03)	0.139
**Paternal BMI**	21.22 (18.71, 22.88)	18.91 (18.07, 20.05)	0.143
**Breastfeeding status**			
Exclusive (n)	12 (40%)	10 (33.4%)	
partial(n)	16 (53.3%)	17 (56.7%)	
none(n)	2 (6.7%)	3 (10%)	
N/A	0	0	
**Pre-intervention anthropometrics**			
WAZ 9mo	-3.75	-3.155	0.060
LAZ 9mo	-2.965	-2.505	0.190
WLZ 9mo	-2.655	-2.33	0.047
**Pre-intervention status n(%)**			
Underweight (WAZ ≤ -2)	29	28	
Stunted (LAZ ≤ -2)	19	20	
Wasted (WLZ ≤ -2)	30	23	
**Pre-intervention markers**			
Hemoglobin (g/dl)	10.5 (9.8, 11.1)	10.3 (8.8, 11.6)	0.986
Pre-albumin (mg/dL)	15.2 (11.7, 16.2)	13.5 (11.55, 15.75)	0.103
Serum AGP (mg/dl)	116 (93, 137)	123 (85, 138.05)	0.895
Serum IGF-1 (ng/ml)	20.13 (10.41, 35.39)	15.81 (11.28, 26.34)	0.360
Serum CRP (mg/dl)	0.118 (0.05, 0.44)	0.24 (0.14, 0.58)	0.166
Serum Ferritin (ng/ml)	21.6 (16, 50)	22 (8, 55)	0.134
Serum Leptin (pg/ml)	129.76 (69.81, 188.56)	180.81 (115.28, 264.97)	0.093
Serum GLP-2 (ng/ml)	1169.89 (976.08, 1607.18)	1101.08 (699.99, 1396.07)	0.175
Fecal MPO (ng/ml)	3034.25 (1250, 7100)	4775 (2250, 10250)	0.551

Fecal NEO (nmol/L)	1675 (850, 2150)	2375 (1525, 5225)	0.004
Fecal Lipocalin-2 (ng/ L)	25.29 (16.16 , 33.20)	28.37 (17.33 , 87.55)	0.008
Urine Claudin-15 (ng/ml)	2.01 (0.92, 2.88)	2.11 (0.82, 2.87)	0.655
Urine Creatinine (μmol/l)	174.32 (92.70, 469.3)	121.52 (91.19, 220.25)	0.168

Supplementary Table 3: Comparison between SEEM cohort and selected cases for this sub-study:

Variables	All Wasted children (n=186) receiving Nutritional Intervention						Controls		
	Responders		p	Non-responder		p			p
	responder in SEEM	with fecal 16S data		non-responder in SEEM	with fecal 16S data		controls in SEEM	with fecal 16S data	
**N**	87	30		99	30		48	28	
**Demographics**									
Gender	59	19	0.66	58	20	0.42	27	18	0.49
Gest age	39 (39 39)	39 (38 39)	0.63	39 (39 39)	39 (39 39)	0.16	39 (39 39)	39 (39 39)	0.44
**Birth anthropometrics**									
WAZ	-2.2 (-3.17,-1.33)	-1.645 (-2.34, -0.96)	0.06	-2.21 (-2.795, -1.655)	-2.09 (-2.69, -1.74)	0.98	-1.03 (-1.585, -0.39)	-1.03 (-1.385, -0.305)	0.75
LAZ	-2.13 (-2.94, -1.15)	-1.51 (-2.14, -0.93)	0.03	-1.83 (-2.63, -1.195)	-1.765 (-2.83, -1.07)	0.96	-0.895 (-1.56, -0.065)	-0.88 (-1.54, -0.16)	0.66
WLZ	-1.26 (-1.96, -0.48)	-1.02 (-1.81, -0.32)	0.52	-1.59 (-2.15, -0.89)	-1.855 (-2.12, -1.17)	0.59	-0.48 (-1.13, -0.095)	-0.445 (-1.155, -0.095)	1.00
**Maternal factors**									
age	29 (24, 35)	30 (24, 38)	0.44	26 (23, 34)	26(23, 35)	0.65	26 (23, 30)	27 (25, 30)	0.65
weight	46 (41.7,51.5)	47 (42.5, 54)	0.42	44.5 (40, 48.79)	45 (40, 48.5)	0.62	49.54 (44.3,53.8)	49.25 (44.5, 53.60)	0.69
height	152.39 (149.75, 155.34)	154.09 (151, 156.58)	0.05	151.80 (147.94, 155)	152.19 (149.5, 156)	0.14	152.05 (148.34, 157)	151.94 (148.25, 155.54)	0.47
BMI	19.73 (18.5,21.7)	19.97 (17.9,22.42)	0.73	19.35 (17.52, 21.3)	18.68 (17.154, 21.02)	0.43	20.99 (19.5,24.28)	20.92 (19.76, 24.46)	0.88
**Paternal BMI**	19.90 (18.71, 22.37)	21.21 (18.71, 22.87)	0.90	19.34 (18.03, 21.75)	18.91 (18.06, 20.04)	0.45	21.85 (19.85, 25.17)	22.45 (20.27, 24.317)	0.73
**Breast feeding status**									
exclusive	38	12		34	10		16	10	
partial	45	16		54	17		28	15	
none	4	2		11	3		3	2	
**Pre-intervention anthropometrics**									
WAZ 9mo	-3.65	-3.75	0.89	-3.28	-3.155	0.76	-0.005	-0.11	0.41
LAZ 9mo	-2.89	-2.965	0.96	-2.49	-2.505	0.67	-0.9	-0.88	0.79
WLZ 9mo	-2.62	-2.655	0.72	-2.37	-2.33	0.76	0.595	0.53	0.59
**Pre-intervention status n(%)**									
Wasted	84	29		94	28		2	0	
Underweight	61	19		63	20		8	5	
Stunted	81	30		82	23		0	0	

Pre-intervention markers

Hb	10.4 (9.3 ,11.4)	10.5 (9.8, 11.1)	0.95	10.4 (8.9, 11.2)	10.3 (8.8, 11.6)	0.91	10.7 (10.15, 11.7)	10.45 (9.5, 11.3)	0.46
Pre-albumin	15.2 (12.9, 16.6)	15.2 (11.7, 16.2)	0.50	13.2 (11.55, 15.75)	13.5 (11.55, 15.75)	0.66	15.3 (13.7, 17.7)	15.2 (13.6, 16.35)	0.17
Serum AGP mg/dl	110.5 (82 ,142.4)	116 (93, 137)	0.58	106.5 (78.5, 138.44)	123 (85, 138.05)	0.65	94.765 (72, 126)	115 (85, 134)	0.16
Serum IGF1 ng/ml	19.06 (10.48, 29.44)	20.13 (10.41, 35.39)	0.56	15.81 (8.87, 24.52)	15.81 (11.28, 26.34)	0.94	27.345 (19.26, 37.65)	24.46 (17.57, 36.48)	0.52
Serum CRP	0.136 (0.048, 0.39)	0.118 (0.045, 0.44)	0.62	0.24 (0.115, 0.64)	0.24 (0.14, 0.58)	0.94	0.0945 (0.05, 0.23)	0.1295 (0.066, 0.368)	0.88
Serum Ferritin	20.5 (7.2, 42.5)	21.6 (16, 50)	0.38	19 8 (05, 39.5)	22 (8, 55)	0.83	9.95 (5.5, 22)	16.95 (5.8, 34.8)	0.36
Fecal MPO ng/ml	2858.25 (1050, 6000)	3034.25 (1250, 7100)	0.59	4933 (1850, 11600)	4775 (2250, 10250)	0.19	4672.25 (2079.5, 10575)	4672.25 (2640.25, 9275)	0.75
Fecal NEO nmol/L	1482.75 (627.5, 2350)	1675 (850, 2150)	0.96	2037.5 (1228.5, 3651.25)	2375 (1525, 5225)	0.20	1633.5 (630, 2750)	1341.5 (485.95, 2600)	0.96
Fecal Lipocalin	20526.15 (12647.85, 32778.5)	25292.58 (16169.55, 33204.25)	0.74	24134.53 (15475.5, 35537)	28370.73 (17331.3, 87550)	0.04	27370 (17347.08, 67500)	40097.925 (20119.5, 94775)	0.29
Urine claudin-15	1.783 (0.93,2.87)	2.01 (0.91,2.88)	0.57	1.4 (0.72,2.66)	2.1065 (0.81,2.86)	0.46	0.909 (0.72,1.13)	0.924 (0.68, 1.15)	0.78
Urine creatinine	139.36 (88.38, 287.24)	174.32 (92.70, 469.3)	0.32	113.80 (68.95, 212.04)	121.51 (91.19, 220.25)	0.61	163.79 (107.98, 205.83)	176.57 (129.65, 212.41)	0.54
Serum Leptin	122.5 (73.22,217.48)	129.76 (69.81, 188.56)	0.57	140.03 (83.68, 217.91)	180.81 (115.28, 264.97)	0.21	279.85 (190.79, 389.19)	258.17 (179.39, 345.51)	0.38
Serum GLP	1288.34 (908.43, 1747.313)	1169.89 (976.08, 1607.18)	0.58	993.43 (723.63, 1350.44)	1101.08 (699.99, 1396.07)	0.95	1705.90 (843.92, 2688.24)	1260.87 (649.51, 2286.14)	0.42

Supplementary Table 4: AchaMum is a locally produced Ready-to-Use Supplementary Food (RUSF) used in Pakistan for treating moderate acute malnutrition (MAM) in children. It was developed by the World Food Programme in collaboration with the Government of Pakistan, often manufactured by companies like Ismail Industries under local fortification standards.

Ingredients:
• Roasted chickpea paste
• Vegetable oil (often palm oil)
• Sugar
• Milk powder
• Vitamins and minerals premix
• Emulsifiers and antioxidants for stability

Composition of AchaMum (per 100g sachet):
•Energy ~520–540 kcal
•Protein ~13–16 g
•Fat ~30–35 g
•Carbohydrates ~45–50 g
•Vitamins & Minerals Fortified with micronutrients, including: Vit A (400 µg), Vit D (5 µg), Vit E, K, B-complex, C (WHO recommendation), Iron (10mg), Zinc (12mg), Calcium, Magnesium and Iodine

Fig S1

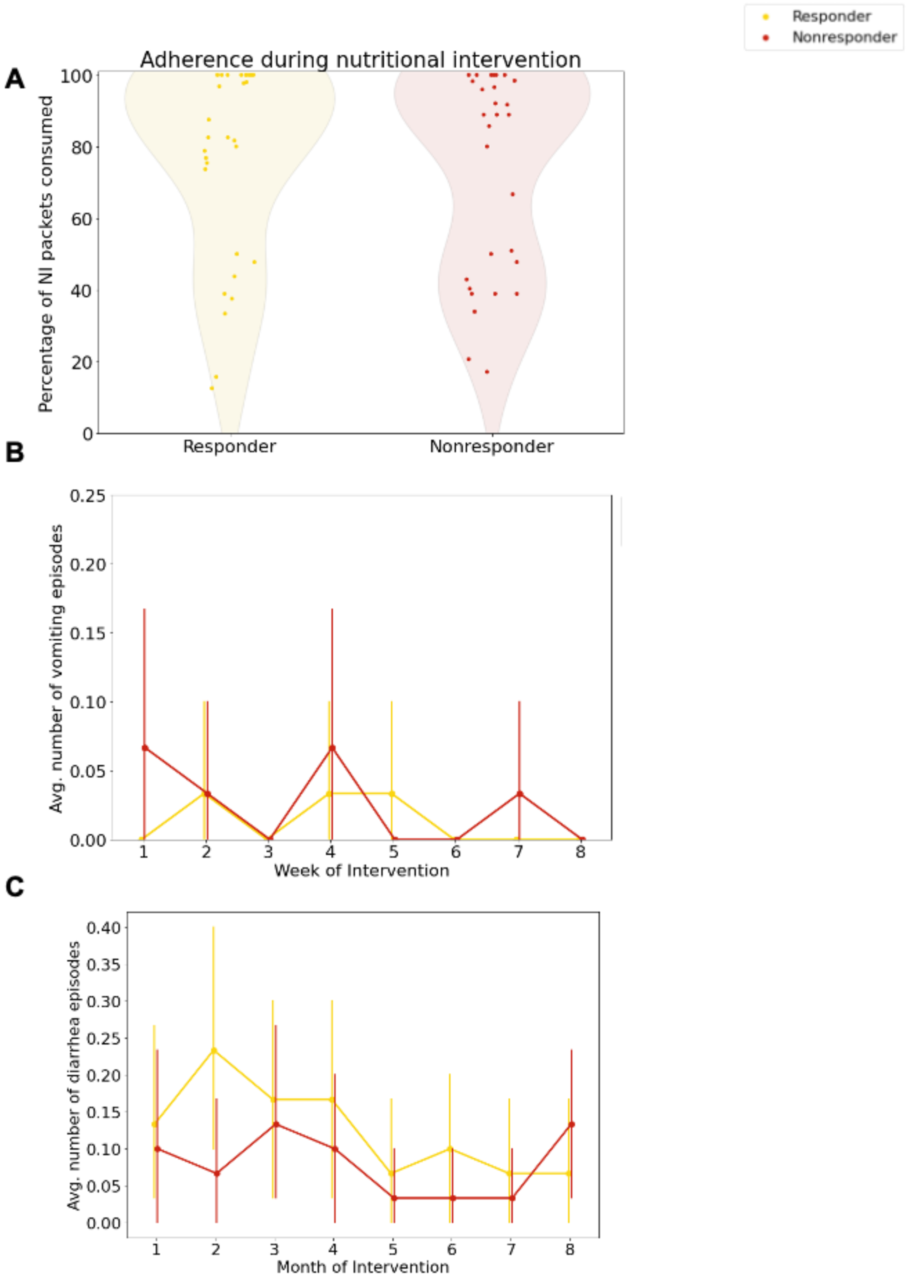

Fig S1: Responders and Nonresponders show similar compliance patterns during the nutritional intervention: (A) The mean number of vomiting episodes per week of the intervention in each group and error bars show standard deviation. **(B)**Mean number of diarrheal episodes per week of the intervention in each group, error bars show standard deviation. **(C)** Adherence to nutritional intervention by the group as shown by the percentage of Acha-mum packets consumed out of the total packets distributed for each child during the intervention.

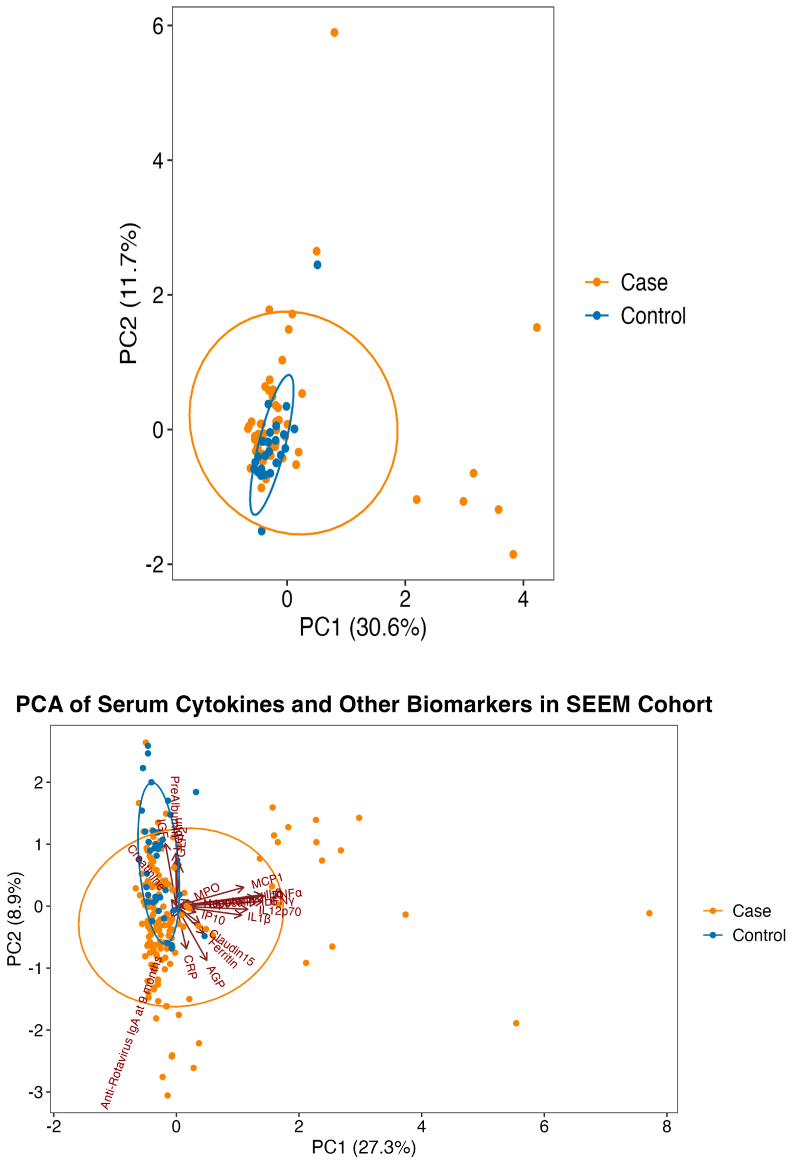

Fig S2: PCA of cytokines in SEEM cohort measured at 9 months colored by nutritional status. Arrows show loadings for each variable. Principal component analysis of serum, urine and fecal biomarker profiles from **(A)** the larger SEEM cohort, wasted children (n=148) and healthy aged-matched children (n=39) and **(B)** wasted cases (n=60) and controls (n=28).

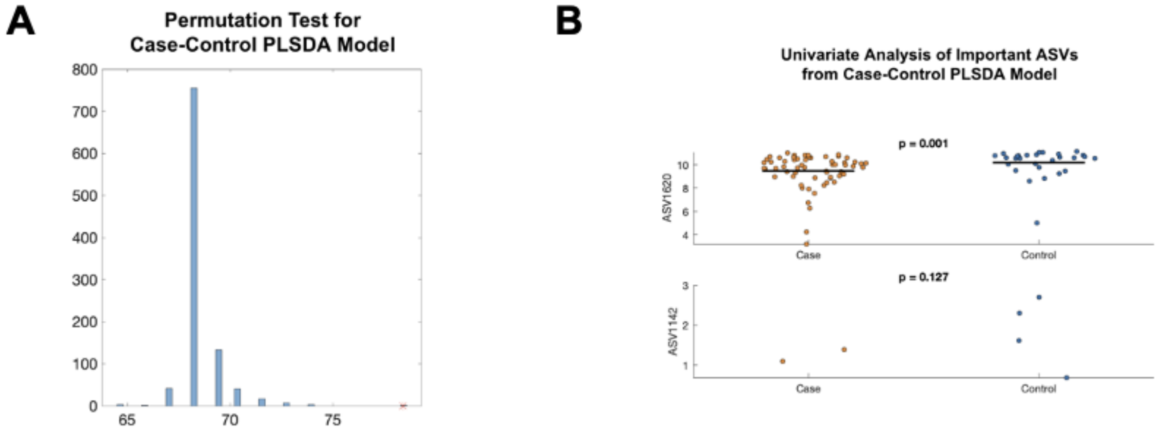

Fig S3: Additional OPLS-DA figures for Fig 5 (A) Results of permutation testing for OPLSDA model in figure 5. The plot shows a histogram of cross-validation accuracy for 1000 randomly permuted models. The correctly labeled model is shown as a red star. (B) Jitter plots showing ASV counts per sample for VIPs identified in OPLSDA analysis.

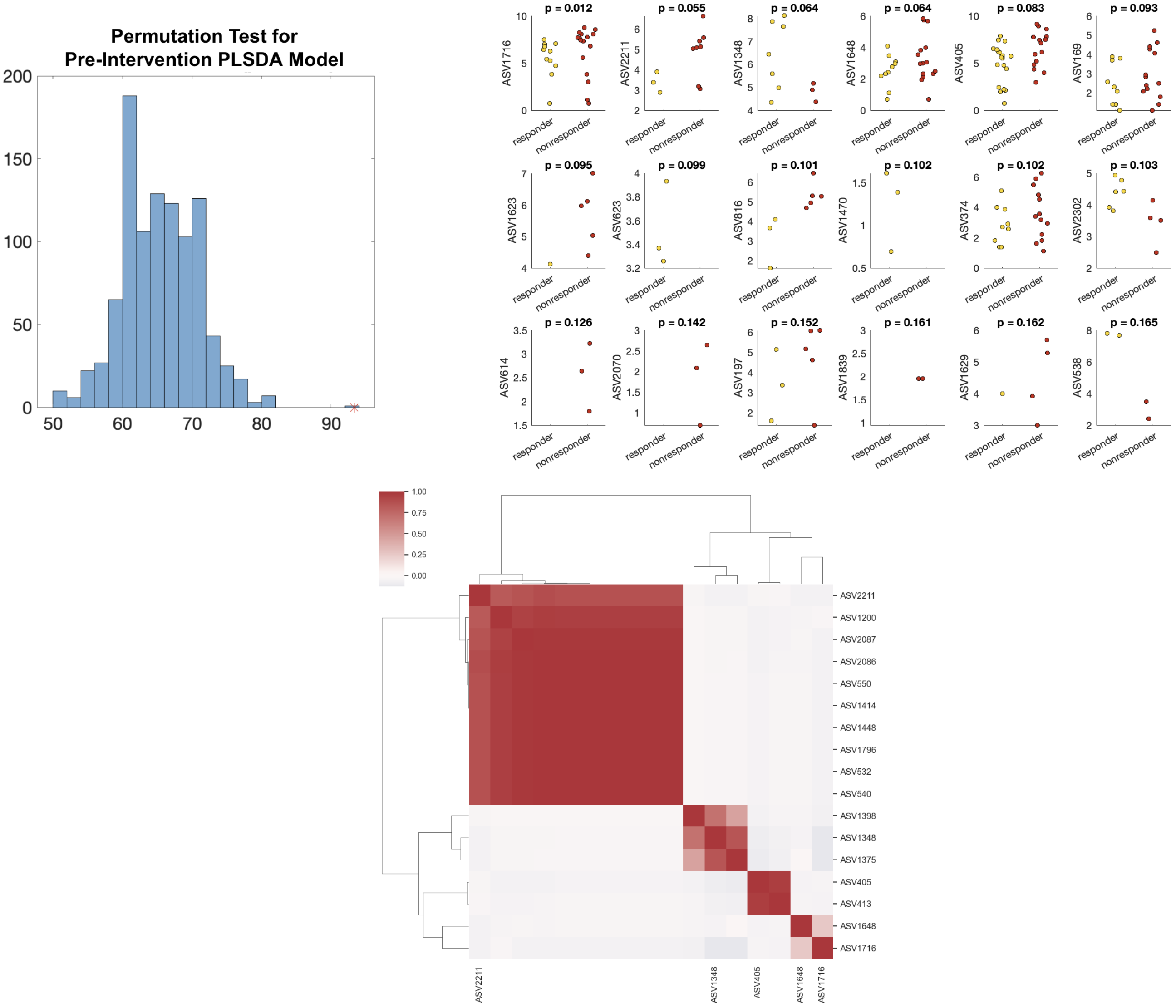

Fig S4: Additional OPLS-DA figures for Fig. 6 (A) Results of permutation testing for OPLSDA model in figure 6. Plot shows a histogram of cross validation accuracy for 1000 randomly permuted models. Correctly labeled model is shown as a red star. **(B)** Jitter plots showing ASV counts per sample for VIPs identified in OPLSDA analysis. **(C)** Heatmap showing a correlation between important ASVs identified in OPLS-DA and other ASVs with >70% correlation that was removed as linearly correlated. The X-axis has only important features labeled, while the y-axis has correlated and important features.

Fig S5

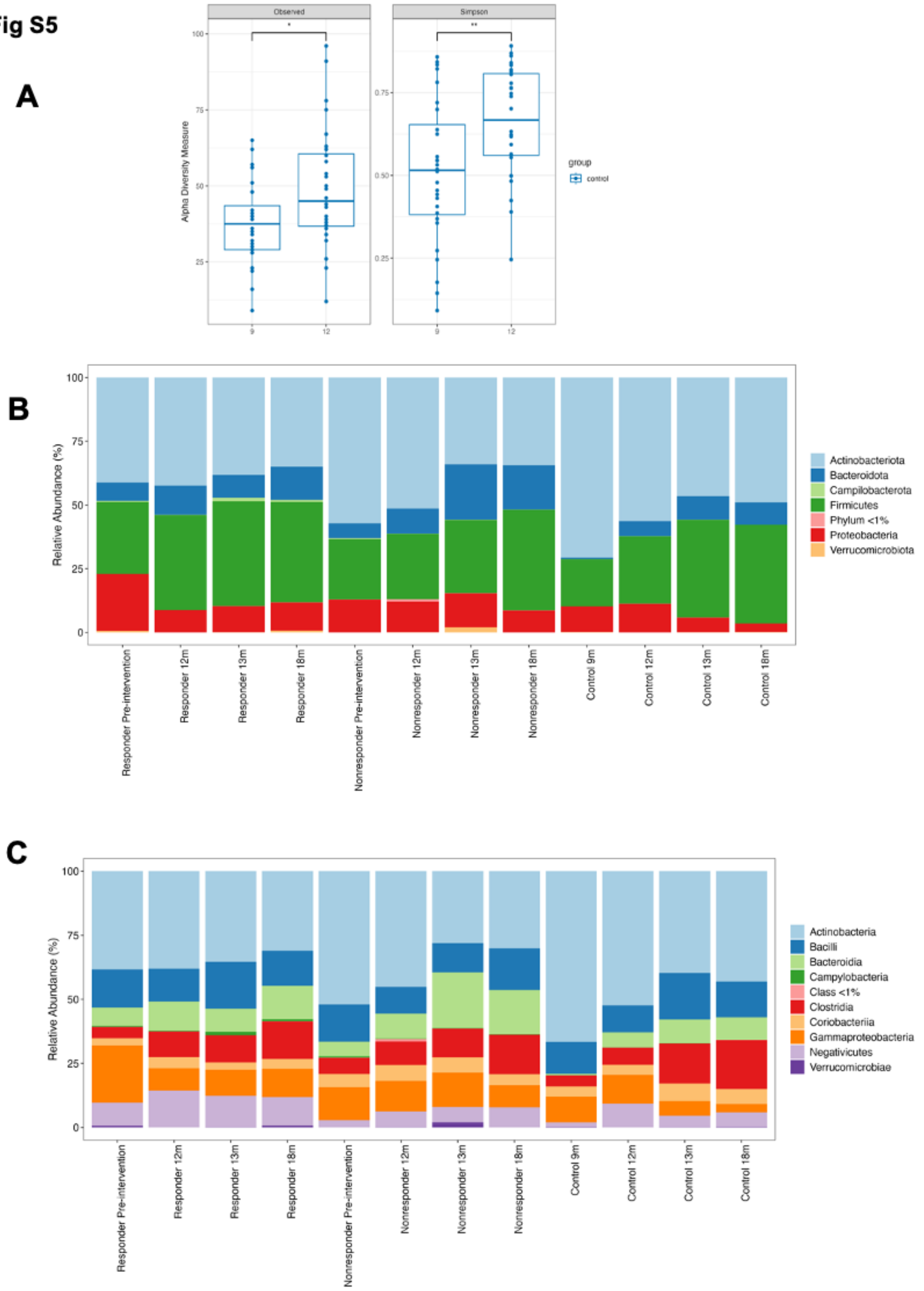

Fig S5: Microbiome shifts during intervention for Fig. 7 (A) Alpha diversity of control samples at 9 months and 12 months of life. (B) Bar plot showing average relative abundance at Phylum level in responders and nonresponders at all timepoints. (C) Bar plot showing average relative abundance at a class level in responders and nonresponders at all timepoints.

Fig S6

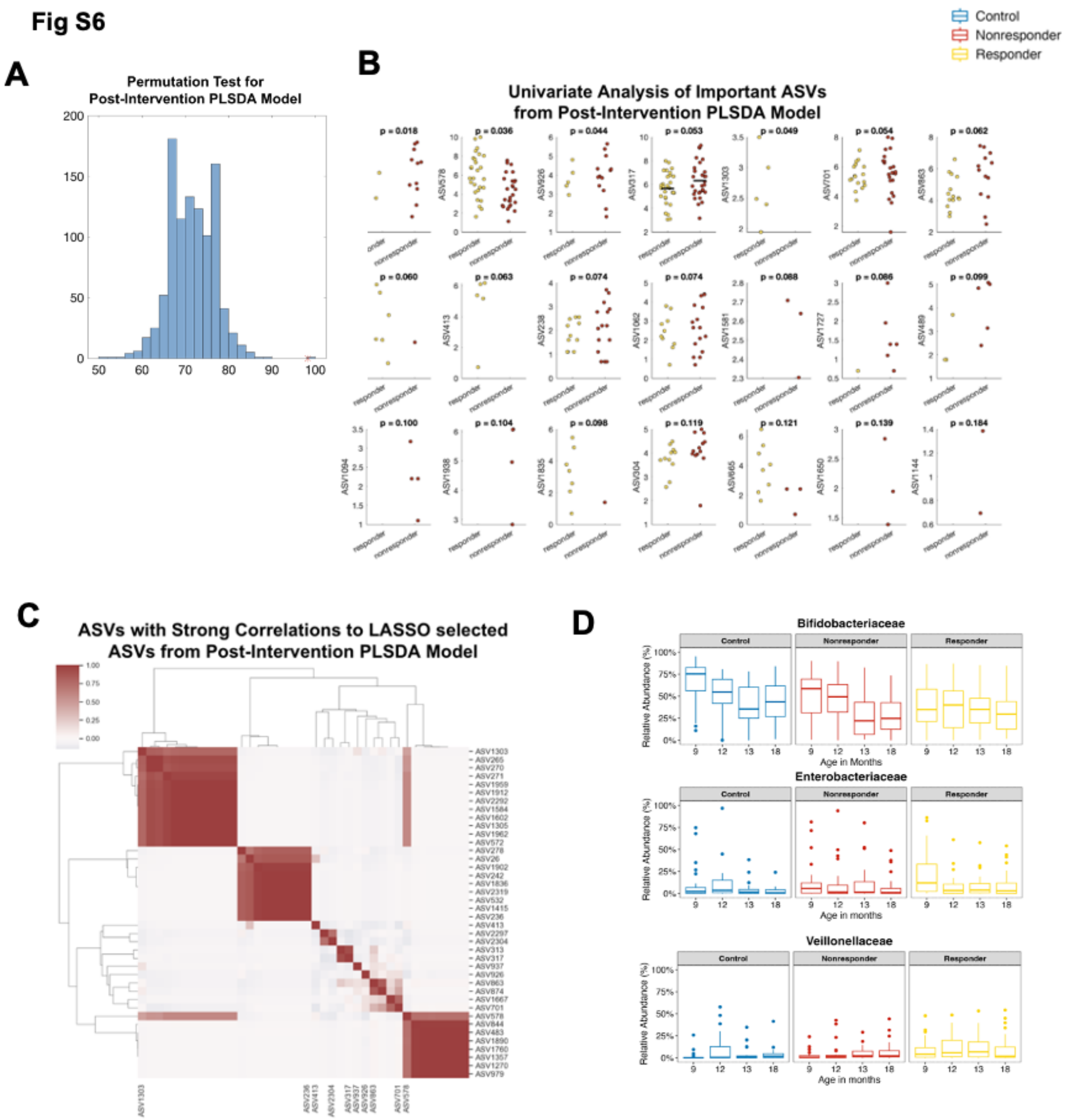

Fig S6: Additional OPLS-DA figures for Fig. 7 (A) Results of permutation testing for OPLSDA model in figure 5. The plot shows a histogram of cross-validation accuracy for 1000 randomly permuted models. The correctly labeled model is shown as a red star. (B) Jitter plots showing ASV counts per sample for VIPs identified in OPLSDA analysis. (C) Correlation network of ASVs with >70% correlation of ASV identified in OPLS-DA. The X-axis has only important features labeled, while the y-axis has correlated and important features labeled. (D) Boxplots showing relative abundance across controls, responders, and nonresponders for 3 bacterial families of interest at all timepoints.

Fig S7

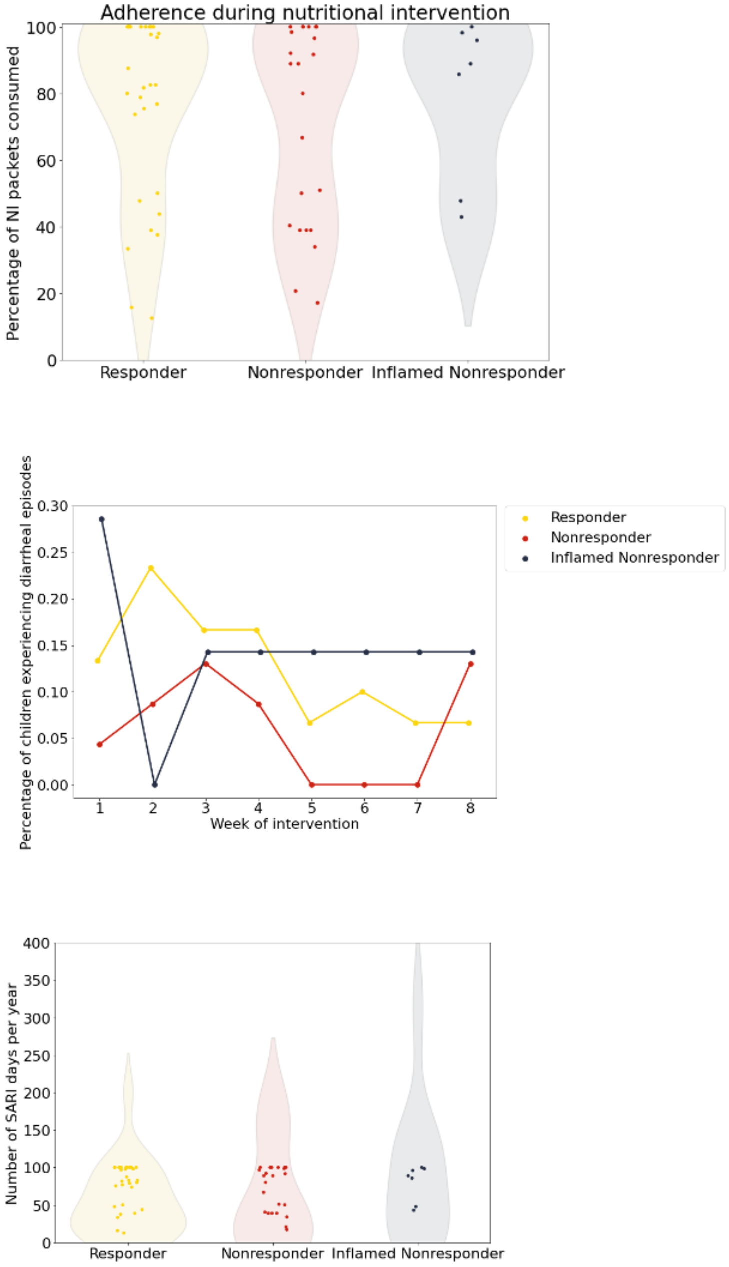

Fig S7: Inflamed nonresponders show similar patterns of adherence and diarrheal episodes but increased respiratory infections: (A) Adherence to nutritional intervention by group as shown by the percentage of Acha Mum packets consumed out of total packets distributed for each child during the intervention. (B) Mean number of diarrheal episodes per week of the intervention in each group, error bars show standard deviation. (C) Jitterplot showing the number of SARI days per year for responders, nonresponders, and inflamed nonresponders.

Fig S8

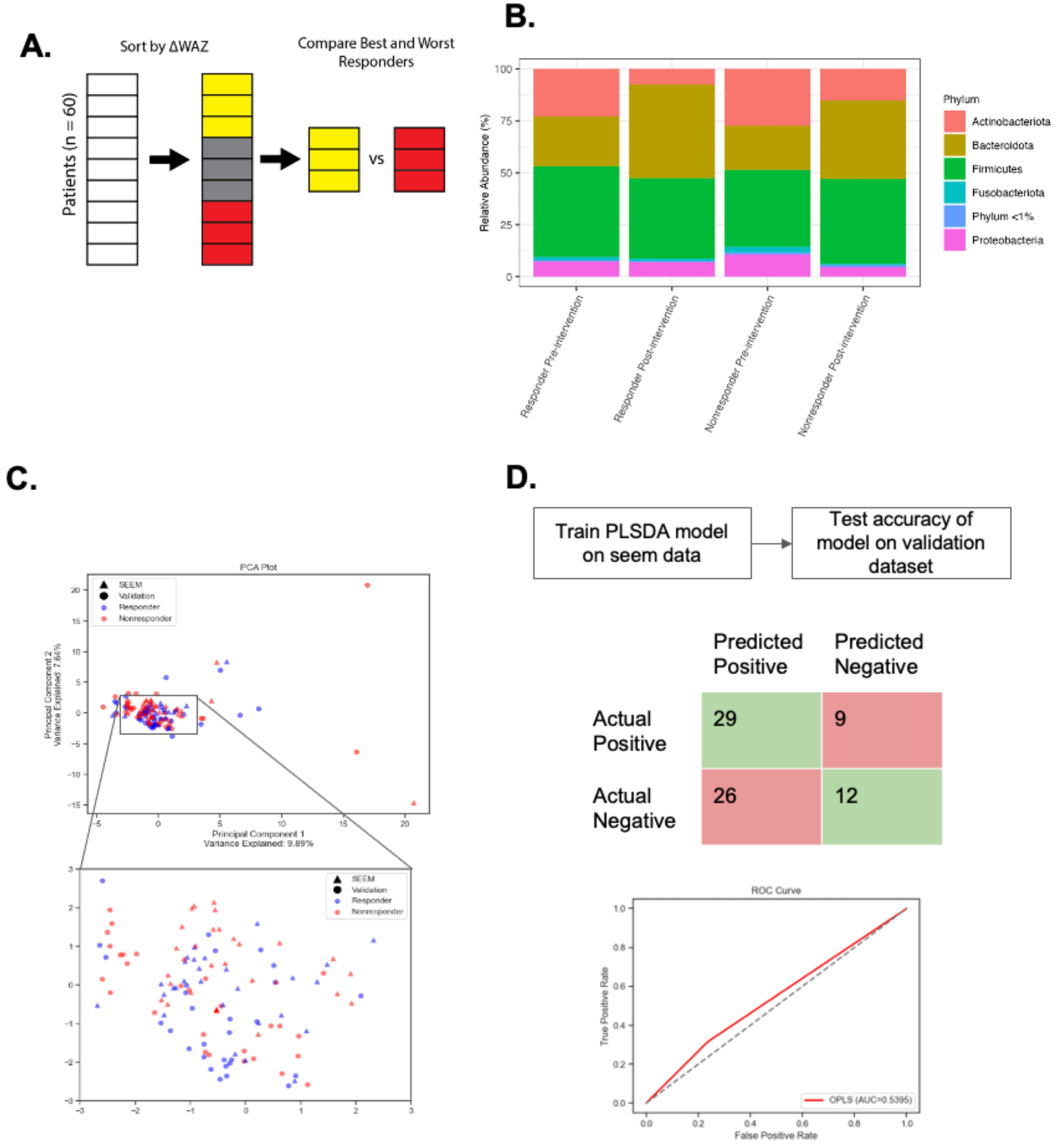

Fig S8: Separate cohort of malnourished children shows different trends in microbiome restructuring during the intervention: (A) Children from the validation dataset who received the chickpea-based intervention were sorted by change in WAZ during the intervention, and the 20

best and 20 worst responders were compared. (B) Bar plots of microbiome composition at pre and post-intervention timepoints in responder and nonresponder groups. (C) Principal component analysis of combined family, class and phylum level microbiome composition data. (D) The PLS model was trained on SEEM data and tested on Chen et al. data. Confusion matrix and ROC curves describe the accuracy of the model.

Fig S9

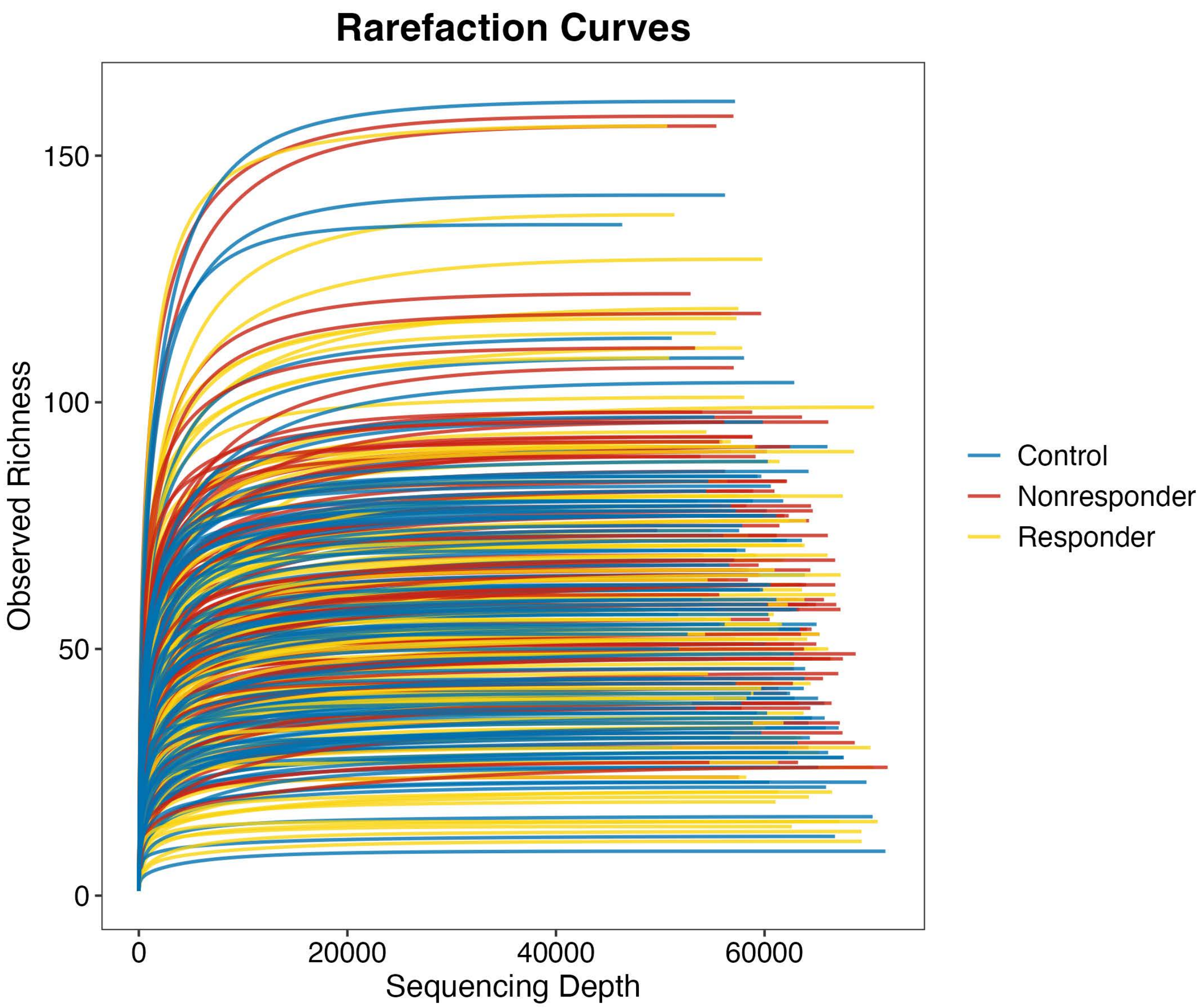

Rarefaction Curves
Observed Richness
0
50
100
150
0
20000
40000
60000
Sequencing Depth
Control
Nonresponder
Responder
